# ^17^O NMR Spectroscopy Reveals CO_2_ Speciation and Dynamics in Hydroxide‐Based Carbon Capture Materials

**DOI:** 10.1002/cphc.202400941

**Published:** 2024-12-16

**Authors:** Benjamin J. Rhodes, Lars L. Schaaf, Mary E. Zick, Suzi M. Pugh, Jordon S. Hilliard, Shivani Sharma, Casey R. Wade, Phillip J. Milner, Gábor Csányi, Alexander C. Forse

**Affiliations:** ^1^ University of Cambridge Yusuf Hamied Department of Chemistry Cambridge CB2 1EW UK; ^2^ University of Cambridge Engineering Laboratory Cambridge CB2 1PZ UK; ^3^ Cornell University, Chemistry and Chemical Biology Ithaca NY 14850 USA; ^4^ The Ohio State University Department of Chemistry and Biochemistry Columbus OH 43210 USA; ^5^ University of California Berkeley Department of Chemical and Biomolecular Engineering and Department of Chemistry Berkeley CA 94720 USA

**Keywords:** Carbon capture, Machine-learning, Metal-organic frameworks, Molecular dynamics, NMR spectroscopy

## Abstract

Carbon dioxide capture technologies are set to play a vital role in mitigating the current climate crisis. Solid‐state ^17^O NMR spectroscopy can provide key mechanistic insights that are crucial to effective sorbent development. In this work, we present the fundamental aspects and complexities for the study of hydroxide‐based CO_2_ capture systems by ^17^O NMR. We perform static density functional theory (DFT) NMR calculations to assign peaks for general hydroxide CO_2_ capture products, finding that ^17^O NMR can readily distinguish bicarbonate, carbonate and water species. However, in application to CO_2_ binding in two test case hydroxide‐functionalised metal‐organic frameworks (MOFs) – MFU‐4l and KHCO_3_‐cyclodextrin‐MOF, we find that a dynamic treatment is necessary to obtain agreement between computational and experimental spectra. We therefore introduce a workflow that leverages machine‐learning force fields to capture dynamics across multiple chemical exchange regimes, providing a significant improvement on static DFT predictions. In MFU‐4l, we parameterise a two‐component dynamic motion of the bicarbonate motif involving a rapid carbonyl seesaw motion and intermediate hydroxyl proton hopping. For KHCO_3_‐CD‐MOF, we combined experimental and modelling approaches to propose a new mixed carbonate‐bicarbonate binding mechanism and thus, we open new avenues for the study and modelling of hydroxide‐based CO_2_ capture materials by ^17^O NMR.

## Graphical abstract

‘^17^O NMR spectroscopy is a developing tool for studying CO_2_ capture in a range of materials. We show that considering dynamics using machine‐learning forcefields provides an effective methodology and is a vital consideration for the accurate modelling of ^17^O NMR spectra. Applying these approaches, we obtain new mechanistic insights in two hydroxide‐based metal‐organic frameworks (MOFs).’

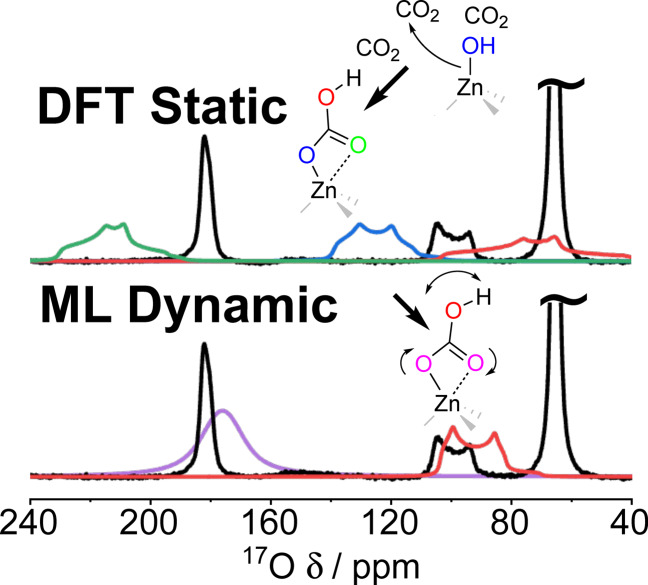

## Introduction

In the context of the global climate crisis, the development of efficient carbon capture materials is gaining urgency. Anthropogenic CO_2_ emissions are the main contributor to increasing atmospheric greenhouse gas concentrations, directly resulting in threatening global temperature rises.^[1]^ In order to limit global warming to 1.5 °C both vast emissions reductions and negative emission technologies are now required.^[2]^ Carbon capture and storage is positioned to play a central roll in both transitioning to zero‐carbon energy sources and as a direct carbon dioxide removal technology to counterbalance residual greenhouse gas emissions. The development of new materials, particularly for direct air capture (DAC), is one of the key areas of research required for achieving economical technological scale‐up. Traditional amine‐based sorbents, used in point‐source capture,^[3]^ suffer from both oxidative degradation and low efficiency regeneration in solvent based systems.[^4^, ^5^, ^6^] Hydroxide‐based solid sorbents, however, are an emerging class of alternative materials, particularly for DAC.[^7^, ^8^] The solid‐state hydroxide chemistries are more robust to oxidative degradation and avoid the corrosion problems associated with solvent‐based amine capture.[^6^, ^7^, ^9^] A growing body of work is focusing on hydroxide‐based materials for DAC,[^8^, ^10^] however, detailed mechanistic information can often be challenging to obtain, hindering further materials optimisation and development.

Metal‐organic frameworks (MOFs) are a well developed and studied class of material for carbon capture applications;[^11^, ^12^, ^13^, ^14^, ^15^, ^16^] in particular, hydroxide‐based MOFs have been demonstrated to achieve high capacities[^17^, ^18^] and high stabilities.[^11^, ^15^, ^16^, ^19^] In most cases a metal‐bound hydroxide will react with CO_2_ to from a chemisorbed metal‐bicarbonate species (equation 1). However, in systems with unreacted adjacent and/or labile hydroxide anions, successive capture (eq. 2) and deprotonation (eq. 3) steps may occur to form free bicarbonate/carbonate chemisorbed species. The interplay of bicarbonate and carbonate species in hydroxide capture is well discussed in the context of humidity‐swing sorbents.[^20^, ^21^, ^22^] Most notably – for all hydroxide‐based sorbents – there is the fundamental stoichiometric difference between bicarbonate (eq. 2) and carbonate (eq. 3) mechanisms, with a bicarbonate product mechanism capturing twice the number of CO_2_ molecules per hydroxide molecule, compared to a carbonate product process. In addition, carbonate mechanisms would likely be subject to slower kinetics due to the multi‐step reaction and the requirement for water dissociation for regeneration.
(1)

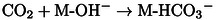



(2)

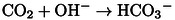



(3)

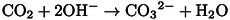




An interesting case study in this regard are *γ*‐cyclodextrin metal‐organic frameworks (CD‐MOFs).[^16^, ^23^, ^24^] These CD‐MOFs have recently been demonstrated to leverage labile hydroxide‐based chemistry to achieve promising CO_2_ capture performance under flue gas conditions.^[16]^ The best performing MOF in this family, KHCO_3_‐CD, demonstrated thermal, oxidative and cycling stabilities along with reasonable capacities for post‐combustion CO_2_ capture (1.43 mmol g^−1^ @ 15 % CO_2_/85 % N_2_, 30 °C and approx. 0.06 mmol g^−1^ @ 0.4 mbar CO_2_, 25 °C). A bicarbonate capture mechanism involving non‐metal bound hydroxide counter ions within the framework pores (eq. 2) was proposed.^[16]^ The free nature of the hydroxide results from the dissociation of the K‐OH bond due to significant hydrogen bonding from the cyclodextrin sugar framework.^[24]^ However, ambiguity remains in the adsorption mechanism with IR, ^13^C NMR and heats of adsorption data unable to fully discard the possibility of carbonate formation (eq. 3). Thus, distinguishing between these two mechanisms is vital for understanding and improving not only CD‐MOF capture materials, but also hydroxide‐based CO_2_ capture systems more generally.

NMR spectroscopy is an established tool for exploring the mechanisms of CO_2_ capture in a large variety of materials.[^25^, ^26^, ^27^] Most NMR studies focus on ^13^C NMR measurements, with ^15^N NMR also utilised in amine‐based systems.[^12^, ^28^] In hydroxide systems, however, such ^13^C NMR data can be ambiguous due to the similar chemical shifts of certain products (e. g. bicarbonate and carbonate) or require further NMR measurements which can introduce their own ambiguity.[^21^, ^22^] Recently, *Berge and Pugh et al*. demonstrated ^17^O NMR spectroscopy as a novel and effective tool for deconvoluting CO_2_ capture mechanisms in amine‐functionalised metal‐organic frameworks.^[29]^ At a cost of enrichment of about £50–75 per sample (20 % atom 17‐oxygen CO_2_), these methods offer great potential for resolving mechanistic ambiguity, giving new insights alongside ^13^C NMR. Particularly, with the quadrupolar nature of ^17^O (I=5/2), additional site‐specific information is accessible from the quadrupolar parameters (C_*Q*_ and *η_Q_*) derived from the observed NMR quadrupolar lineshapes: C_*Q*_, characterising the magnitude of the quadrupolar interaction (expressed in the linewidth), and *η_Q_*, the asymmetry of the interaction (expressed as lineshape geometry) – see SI methods *NMR Spectroscopy*.

Due to the relative scarcity of literature and the uniqueness of individual systems, making assignments of ^17^O NMR spectra for hydroxide‐based systems can be challenging. As such, computational methods, in particular density functional theory (DFT), provide valuable tools in aiding assignments of the observed spectra.[^30^, ^31^, ^32^] In this study, we first demonstrated static DFT results to establish the expected ^17^O NMR parameters for hydroxide‐based CO_2_ capture. However, the utility of these results is shown to be limited in this context and we thus stress the importance of including dynamic effects in computational NMR modelling for these systems. By training a machine‐learning force field (MLFF)[^33^, ^34^, ^35^, ^36^] on DFT data, we are able to run molecular dynamics at ab‐initio accuracy with reduced computational cost for NMR parameter prediction.[^37^, ^38^, ^39^] Fast dynamics on the time scale of nanoseconds are well described by molecular dynamics (MD) trajectories, however, slower motions have previously been required to be assessed by further NMR measurements or be parameterised by fitting of spectral data to infer dynamic processes.[^40^, ^41^, ^42^, ^43^] Notably, this often leads to continuum motions, e. g. molecular wobbling, being approximated by discrete hop models.[^40^, ^43^] In this work, we overcome this problem by directly combining MLFF‐MD and stochastic intermediate exchange methods.[^44^, ^45^] We thus propose a novel approach for including dynamic NMR effects in fast, intermediate and combined regimes in a pre‐determined – rather than empirically derived – fashion. This completes a universal approach for utilising machine‐learning force fields for computational NMR prediction, across a range of chemical exchange regimes.

We demonstrate this combined computational and ^17^O NMR approach on the well‐studied MFU‐4l MOF.[^15^, ^19^] The established metal‐binding mechanism (eq. 1) in MFU‐4l is refined to provide precise insights into the dynamics of the bound bicarbonate species. Then, through application to KHCO_3_ and K_2_CO_3_ ⋅ 1.5 H_2_O solid powders and using additional MLFF molecular dynamics,^[46]^ we are able to elucidate an improved, mixed bicarbonate‐carbonate (eq. 2/3) capture mechanism for KHCO_3_‐CD‐MOF using ^17^O NMR spectroscopy.

## Results and Discussion

### ^17^O NMR Parameters of CO_2_ Species Captured by Hydroxide

**Figure 1:**
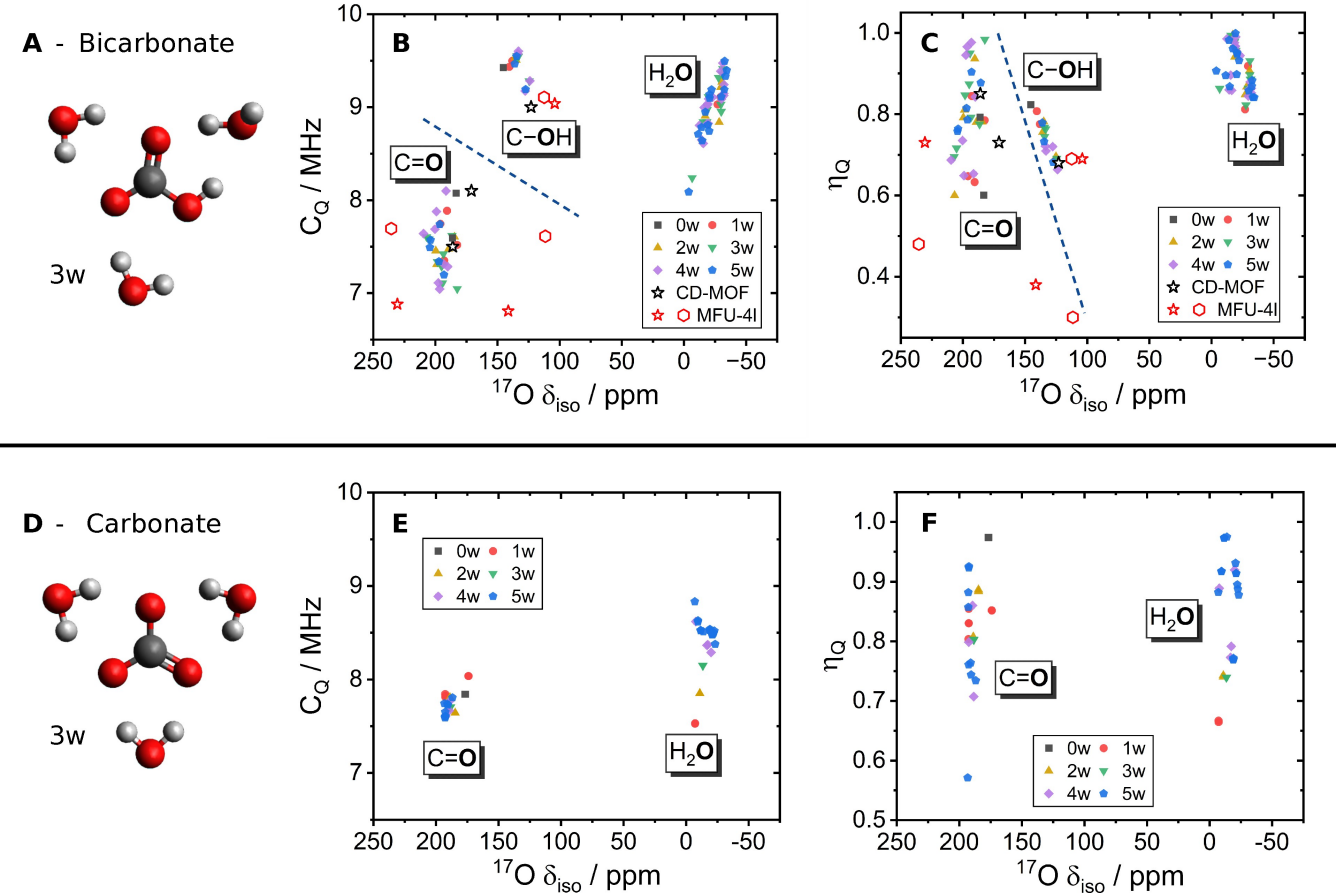
Representative DFT calculations resolve speciation differences through ^17^O NMR parameters. Calculated parameters for hydrated bicarbonate anion clusters (A–C) and carbonate anion clusters (D–F) with varying degrees of hydration from 0 to 5 water molecules (0‐5w). Calculated quadrupolar NMR parameters *C_Q_* (B, E) and *η_Q_* (C, F) are plotted against isotropic chemical shift. Blue dash lines act as guidelines to show the divisions of the different environments. Equivalent *C_Q_* vs *η_Q_* comparisons are plotted in Figs. S3‐S4. MOF parameters of KHCO_3_‐CD‐MOF (black star, B, C) and two binding modes of MFU‐4l (red: mode A – star, mode B – hexagon) are also included (structures see Figure S10 and S11).

Investigating CO_2_ capture by hydroxides through ^17^O NMR spectroscopy requires the establishment of the expected range of quadrupolar NMR parameters for the predicted products of capture, i. e. bicarbonate and carbonate. Representative hydrated anion clusters of bicarbonate (Figure 1A–C) and carbonate (Figure 1D–F) were generated using static DFT calculations in line with those previously reported in the literature (see Figures S1‐S2 for full structure list).[^47^, ^48^, ^49^, ^50^] These structures were identified as the most general candidate structures with their diversity of hydrogen‐bonded oxygen environments hypothesised to represent the likely environments found in the binding sites of solid‐state CO_2_ sorbents. For comparative purposes, calculated NMR parameters for periodic boundary condition structures of bound CO_2_ in KHCO_3_‐CD‐MOF (Figure S11) and two modes proposed for MFU‐4l (Figure S10) are also included in Figure 1.

For the bicarbonate clusters, carbonyl oxygens are clearly distinguished at high *δ_iso_* =174–209 ppm, above that of hydroxyl oxygens at *δ_iso_* =125–145 ppm and water *δ_iso_* =−4 to −34 ppm (Figure 1B−C). Additionally, the C_*Q*_ values of hydroxyl oxygens are found to be higher, C_*Q*_=9–9.6 MHz, than for carbonyls, C_*Q*_=7–8.1 MHz. Comparison to the MOF structures also corroborates these trends with the hydroxyl matching in all three NMR parameters. The carbonyls are more spread for MFU‐4l with respect to *δ_iso_* and *η_Q_*, though C_*Q*_ remains in agreement. This is due to polarisation induced by the Zn‐metal centre, whereas the CD‐MOF remains in agreement with the clusters in both *δ_iso_*, *η_Q_* and C_*Q*_ as no metal‐bicarbonate bond is present.

Notably, the hydroxyl environments in bicarbonates are thus predicted to be readily distinguished in an experimental spectrum (and additionally from any H_2_O oxygens present), allowing for bicarbonate and carbonate products to be distinguished. The *η_Q_* values for all environments are in the range ≈0.3–1, making any inferences for assignments from these relatively broad distributions challenging.

Comparing carbonate and bicarbonate anions, the chemical shift range for carbonate carbonyl oxygens is *δ_iso_* =174–193 ppm (Figure 1E–F). This is overlapping with the carbonyl range of bicarbonate anions, although is skewed to the lower ppm values of the distribution (see Figures S5‐S6). Likewise, the carbonate C_*Q*_ values are found at the higher end of the bicarbonate range, C_*Q*_=7.6–8 MHz, though there remains significant overlap (see Figures S5b‐S6b). This result from static DFT that bicarbonate and carbonate carbonyl environments are indistinguishable with respect simply to their ^17^O NMR parameters is a somewhat unsurprising result given their chemical similarity. However, the clear separation of hydroxyl groups gives strong potential for assigning speciation of CO_2_ capture products. The NMR parameters of the structures used in Figure 1 were also calculated using the 6‐311+G(d,p) basis‐set of the same level of theory (triple *ζ*‐level),^[51]^ no significant changes in parameter values were observed (see Figures S5‐S9).

### ^17^O NMR Spectroscopy – Functional Group Assignment

**Figure 2:**
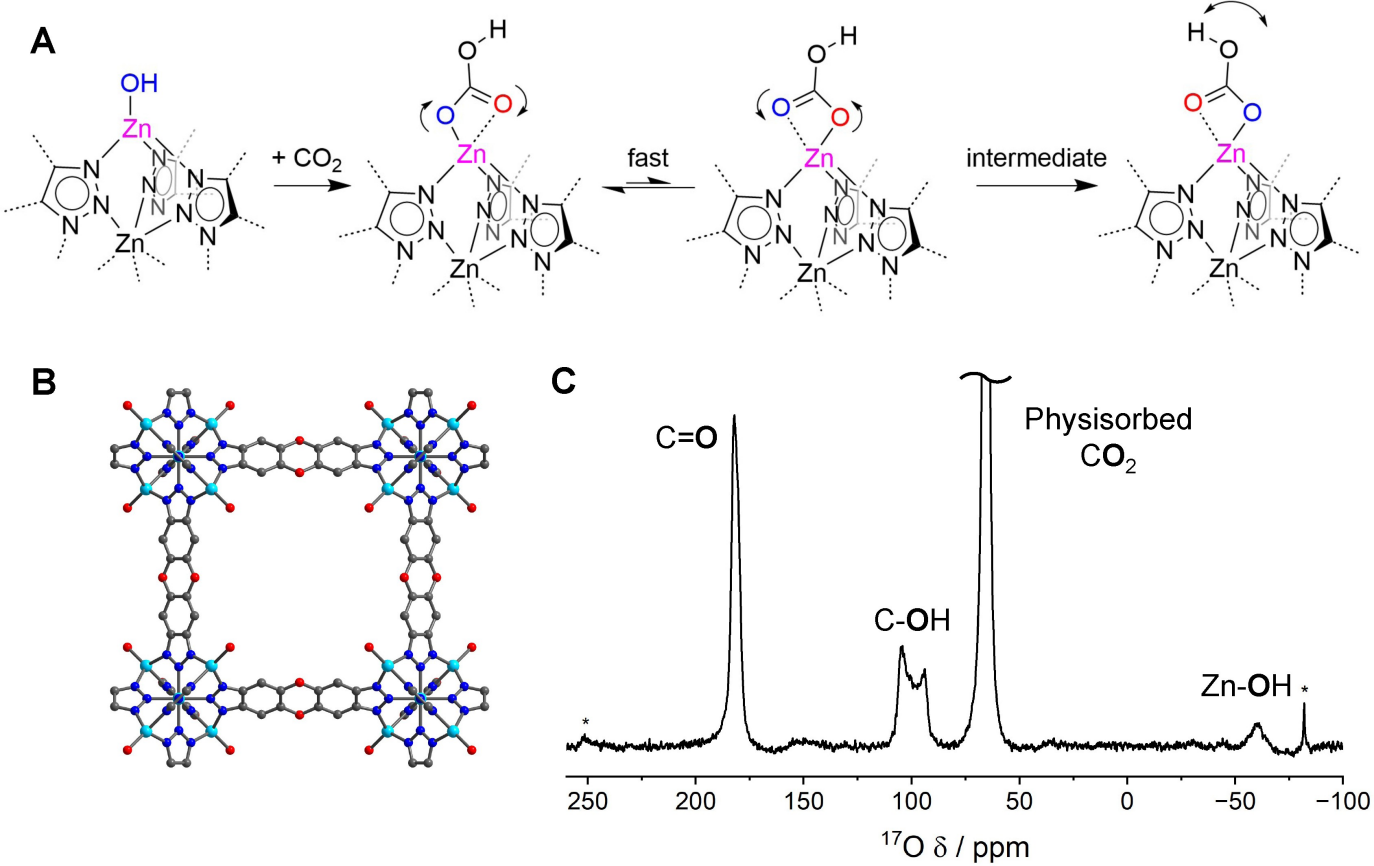
The ^17^O NMR spectroscopy and structures of MFU‐4l. (A) A schematic showing the CO_2_ binding and potential dynamics at the Zn‐OH binding site – blue and red denote distal and proximal carbonyls (relative to the hydroxyl proton), respectively. (B) A representation of the crystal structure of MFU‐4l with the Zn‐OH moieties pointing into the pore cavity (Zn – light blue, O – red, C – grey, N – dark blue). (C) The experimental ^17^O NMR spectrum (23.5 T, 20 kHz MAS, 10.8 h, hahnecho) of MFU‐4l dosed with 0.8 bar C^17^O_2_. * Denotes spinning side bands.

With the expected theoretical parameters established, the application of ^17^O NMR spectroscopy to hydroxide‐based CO_2_ capture materials was initially assessed using the well‐known MFU‐4l (Zn_5_(OH)_4_(btdd)_3_) MOF, Figure 2.[^15^, ^18^] MFU‐4l has well defined, isolated monodentate Zn‐hydroxide sites situated within cubic pores (Figure 2A–B).[^15^, ^19^] These hydroxide sites are thought to react in a 1 : 1 ratio with CO_2_ to form individual bicarbonate type structures via an insertion mechanism.^[15]^ The ^17^O NMR spectrum of a C^17^O_2_ dosed sample of the MOF showed 4 clear resonances (Figure 2C). Physisorbed CO_2_ at 68 ppm is readily assigned from previous literature.^[29]^ Both the carbonyl, 175–178 ppm, and hydroxyl, 90–110 ppm, environments are assigned initially in comparison to the predicted shifts for bicarbonate clusters in Figure 1. Finally, the peak at −50 to −70 ppm is assigned to unreacted Zn‐hydroxide site, in agreement with literature.[^52^, ^53^] The clear identification of carbonyl and hydroxyl functional groups (at an approximate 2 : 1 ratio, see S12) is in agreement with the accepted bicarbonate binding structure in MFU‐4l and gives promise to ^17^O NMR as a technique for making speciation assignments.

It is noted that for this final assignment, although some Zn‐OH sites remain apparently unsaturated at 0.8 bar CO_2_, the appearance of signal (given the low natural abundance of ^17^O) must be a result of reaction and exchange with the ^17^O isotope labels in the dosed CO_2_. This indicates that there is enrichment through dynamic bond forming and breaking at some Zn‐OH sites that do not strongly bind CO_2_. This supports previous observations that binding of sequential equivalents of CO_2_ leads to weaker binding energies.^[15]^

Although the chemical shift of the carbonyl peak agrees well with the cluster DFT calculations in Figure 1, comparison to the calculated MFU‐4l values is poor. The asymmetric Zn‐oxygen bonds (Figure 2A) predicted by DFT,^[18]^ and resulting asymmetric polarisation, leads to two calculated C=O peaks separated by 90–120 ppm (Figure 1B–C), with Zn‐binding leading to lower chemical shifts. Additionally, the narrowness of the observed peak indicates that the full quadrupolar lineshape is not being expressed. A quadrupolar lineshape fitting for the C=O peak returns a C_*Q*_=2.7 MHz, much lower than the static DFT predictions (Figure 1B). Similarly, although a quadrupolar lineshape is expressed for the hydroxyl peak, the fitted C_*Q*_=5.5 MHz is lower than the DFT predictions of 9.0–9.1 MHz.

These phenomena are most likely explained by some degree of dynamic motion (e. g. Figure 2A) partially averaging some of the quadrupolar interaction, and providing chemical equivalence of the two carbonyl environments. In order to investigate this further and produce a quantitative model for the NMR spectrum, detailed molecular dynamics simulations with a machine‐learning force field were performed.

### Machine‐Learning Force Fields (MLFFs) for Dynamic ^17^O NMR Peak Prediction

Ab‐initio molecular dynamics is a well established approach for capturing dynamic effects in the simulation of NMR spectra,[^54^, ^55^, ^56^, ^57^, ^58^, ^59^, ^60^, ^61^, ^62^, ^63^, ^64^, ^65^, ^66^, ^67^, ^68^, ^69^] including in the context of CO_2_ diffusion and physisorption materials.[^40^, ^41^, ^43^] MD has been used to gain qualitative insights and to sample relevant 3D configurations which can be used to compute an ensemble of NMR parameters for each nucleus. However, the computational cost of direct ab‐inito MD severely limits this approach in terms of simulation time and system size. The cost can be circumvented through the use of machine‐learning force fields, which are trained on a set of ab‐initio reference calculations, such as DFT, and then used to predict energies and forces on unseen configurations at near ab‐initio accuracy.[^33^, ^34^, ^35^, ^36^, ^70^, ^71^, ^72^, ^73^]

Even with the computational speedup that MLFFs offer, these approaches only capture fast NMR regime dynamic processes at around nano‐second time‐scales.[^37^, ^38^, ^39^] However, NMR lineshapes can be affected by changes to the chemical environment at much longer timescales. Indeed, the effect of a dynamic processes on the observed NMR spectra depends significantly on the relationship of the exchange rate, *k*, and the resonant frequency difference, Δ*ν*, between the initial and final states. For quadrupolar nuclei such as ^17^O (I=5/2) in the solid state, this Δ*ν* importantly differs from the more classic solution‐state case where Δ*ν* is simply the frequency difference of the isotropic chemical shifts. Here, Δ*ν* has anisotropic contributions from the second‐order quadrupolar interaction (even under MAS conditions), including tensor orientation, thus providing an analytic definition of Δ*ν* can be challenging.^[17]^ However, the interpretation of interconversion of resonant frequencies, now including chemical shift and quadrupolar interaction contributions, with respect to exchange rates and the effects on NMR spectra remains analogous to the classical solution state case.[^75^, ^76^, ^77^]

**Figure 3:**
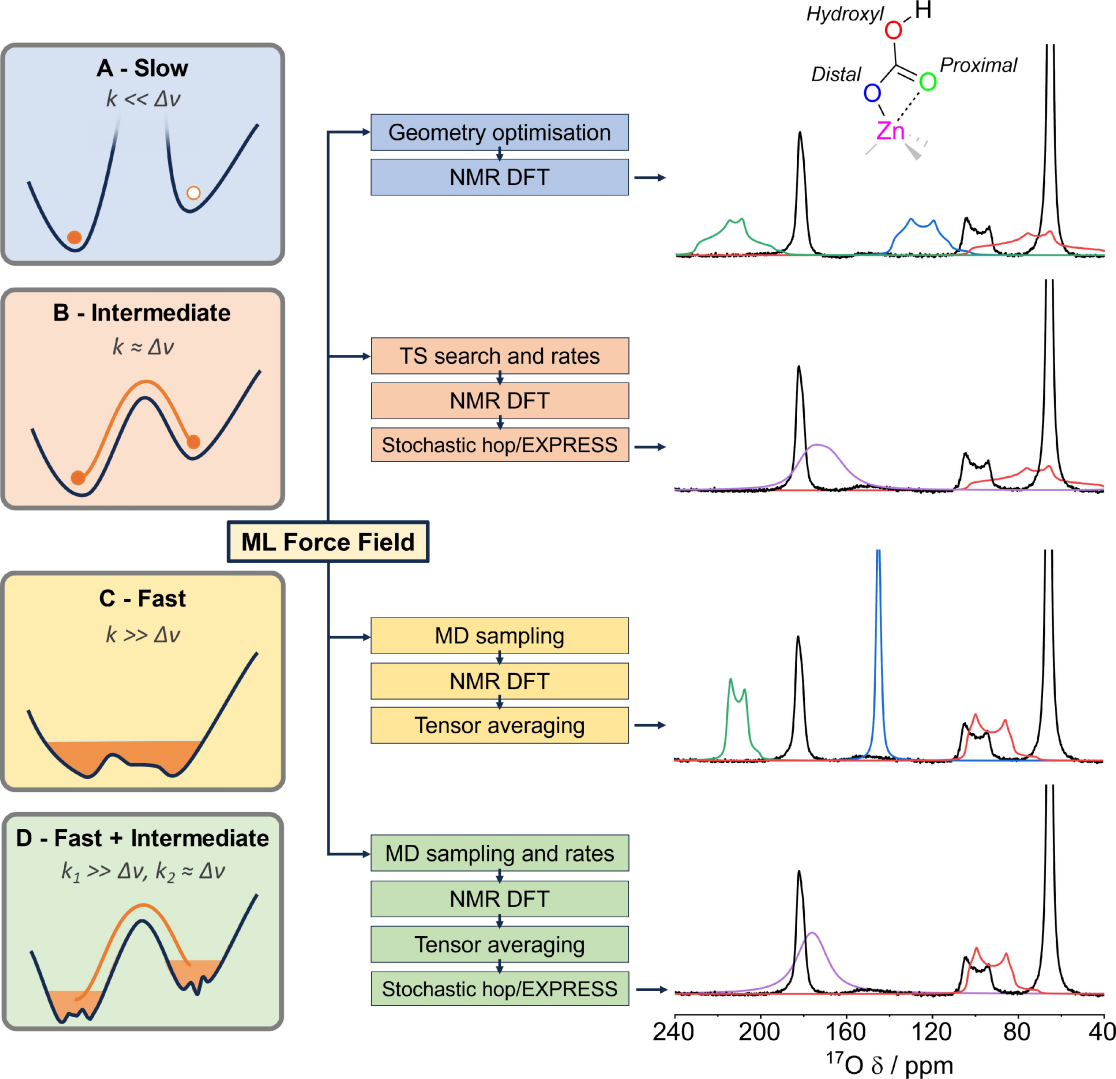
Capturing dynamic effects from fast to slow chemical exchange regimes using ML force fields. The experimental ^17^O NMR spectrum (right, black) of MFU‐4l (23.5 T, 20 kHz MAS, 10.8 h, hahnecho) dosed with C^17^O_2_ modelled in four different exchange regimes. Simulated peak environments are denoted as red – hydroxyl, green – proximal carbonyl, blue – distal carbonyl, and purple – averaged carbonyls with proton hop. Schematics of the exchange regimes and workflows utilising ML force‐fields are displayed (left). Panel D demonstrates the most accurate model combining both fast (k1 represents the exchange rate within the potential well) and intermediate (k_2_ represents the exchange rate between the potential wells) exchange modelling.

Here we consider three separate chemical exchange regimes: slow exchange (k< <Δ*v*), where each environment is well resolved in the NMR spectrum; fast exchange (k> >Δ*v*), where rapid dynamics lead to a complete averaging of individual NMR environments; and intermediate exchange (k≈Δ*v*) when NMR environment coalescence is observed but averaging is only partial. In the case of CO_2_ binding in MFU‐4l, it is clear from static DFT models, Figure 3A (NMR parameters from Figure 1, red star values are taken), that a slow exchange or static regime is inaccurate.

Therefore, we propose a comprehensive approach that is able to capture dynamic effects across multiple chemical exchange regimes (Figure 3). By sampling rare events with both MD and transition state searching we obtain effective isotropic and anisotropic ^17^O NMR tensors and lineshape simulations. To facilitate accurate sampling and the calculation of transitions states we train a ML force field to near ab‐initio accuracy. The MLFF training follows the active learning approach detailed in Reference [78].^[78]^ These methods allows us to deconvolute the multi‐layered dynamics in the example case of MFU‐4l MOF:

***Fast regime***. We use direct MLFF molecular dynamics to capture fast processes. The final spectrum, Figure 3C, is obtained by point wise averaging of the individual DFT shielding and EFG tensors across 18 samples of the trajectory. For the MFU‐4l MOF we sample along a 2 ns trajectory, thus any motion observed is in the fast exchange regime (k≥500 MHz). The MD reveals a well defined asymmetric carbonyl ‘seesaw’ motion (Figure 2A, step 2 – see supporting information.mov file). Notably, the hydroxyl proton was not seen to move significantly leading to two chemically distinct carbonyl sites, one proximal to the proton (Figure 2A – red) and one distal to the proton (Figure 2A – blue). This ‘seesaw’ motion was found to be asymmetric at a ratio of 83 : 17 in favour of the distal carbonyl being bound to the Zn (Figure S13–S14). This is hypothesised to be due to a weak interaction between the proximal carbonyl and the hydroxyl proton, with the resulting polarisation leading to a reduction in relative binding strength to the Zn. Compared to the slow regime, there is marked narrowing of all three environments as would be expected from the partial motional averaging of the quadrupolar interaction. The hydroxyl peak (Figure 3C – red) now matches well in shape to the experimental spectrum with only a Δ*δ_obs_* ≈5 ppm discrepancy compared to experiment. However, the distinct proximal and distal carbonyl environments are still well resolved and require further consideration.

***Intermediate regime***. To capture longer timescale events we do an extensive local minima energy structure search using minima hopping.^[79]^ We find the exchange rates connecting minima using a transition state search with the MLFF. For MFU‐4l, a rotational flip of the hydroxyl group of the bicarbonate (Figure 2A) is found which leads to the two carbonyls becoming chemically equivalent (see S15). We use the nudged elastic band method and find an energy barrier of 0.49 eV, which corresponds to a rate of 2.4×10^5^ Hz under the harmonic approximation at room temperature (see S16). Using the calculated rate constant along with the static DFT carbonyl values (see Figure 1A–C) as inputs to the stochastic hopping package EXPRESS,^[44]^ the two distinct carbonyl peaks coalesce, Figure 3B. Variable temperature NMR (Figure S17) identifies significant broadening at lower temperature supporting this proposed hydroxyl proton flip is at the fast exchange edge of the intermediate regime (*k*≈Δ*v*). This intermediate exchange model gives a clear improvement to the carbonyl lineshape with a single coalesced peak differing in Δ*δ_obs_* ≈13 ppm to experiment, however, an excessive peak width remains (C_*Q*_=6.2 MHz, c.f. experimental C_*Q*_=2.7 MHz).

***Fast + Intermediate regime***. To combine the effects of fast and intermediate exchange processes, we use EXPRESS to model a stochastic hopping on the fast regime MD averaged tensors from Figure 3C. Modelling both the effects of the ‘seesaw’ and proton hop motions results in good agreement with experiment, Figure 3D. This novel combination of dense sampling MD averaging and discrete site hops provides the most accurate model for both the carbonyl, with only a Δ*δ_obs_* ≈6 ppm discrepancy and a peak width more accurately described by a C_*Q*_=4.2 MHz, and hydroxyl peaks, modelled analogously to Figure 3C. In the intermediate regime observed for the carbonyl, there is a degree of sensitivity of the simulated peak shape to the precise value of the calculated rate constant (see Figure S18). Such sensitivity explains the discrepancy in the final carbonyl model, however, overall, the success of this modelling supports a specific two‐component motion of carbonyl ‘seesaw’ and proton hopping in the CO_2_ binding mode in MFU‐4l (Figure 2A). This reveals not only the detailed nature of CO_2_ binding in MFU‐4l but also provides insight that can be transferred to other similar metal‐binding systems.^[17]^

The MLFF is highlighted as critical in keeping the computational cost affordable when modelling dynamics effects across all exchange regimes. Without the use of MLFF‐MD, the nature of dynamics and exchange regime would be unknown; rate constant information would have to be assumed for stochastic hop modelling, and no refinement of the non‐explicitly exchanging environments (e. g. hydroxyl peak) would be possible. Notably, this contrasts significantly to the ^17^O NMR modelling of amine‐based carbon capture materials, where static DFT modelling is sufficient.^[29]^ Thus, we demonstrate that the modelling of dynamics by MLFF methods is a key component for the assessment of hydroxide‐based CO_2_ capture materials.

### New Insight into the Carbon Capture Mechanism of KHCO_3_‐CD‐MOF

The successful modelling of CO_2_ binding in MFU‐4l by a combination of ^17^O NMR and MLFF‐MD averaging methods establishes a clear set of methodologies for studying hydroxide‐based CO_2_ capture. KHCO_3_‐CD‐MOF is a promising CO_2_ capture sorbent,^[16]^ however, mechanistic uncertainty between bicarbonate and/or carbonate chemisorption product(s) remains which ^17^O NMR methods are well placed to resolve.

**Figure 4:**
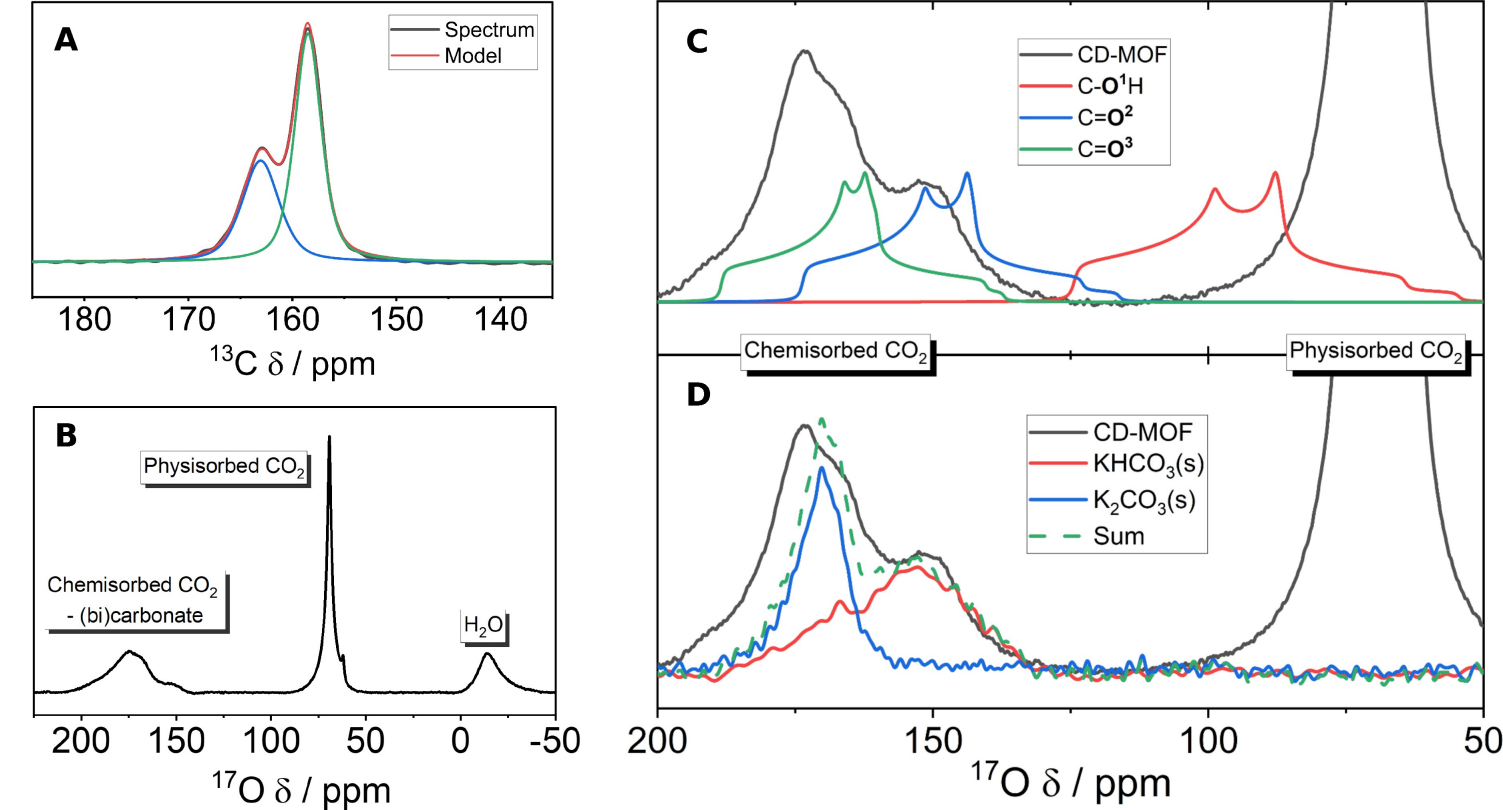
The NMR spectra and assignments of CO_2_ dosed KHCO_3_‐CD‐MOF. NMR spectra of KHCO_3_‐CD‐MOF dosed with ≈1 bar enriched CO_2_: (A) ^13^C (9.4 T, 12.5 kHz MAS, 1.7 h, CPMAS) and (B) {^1^H}^17^O NMR spectra (23.5 T, 20 kHz MAS, 16.2 h, one‐pulse) of KHCO_3_‐CD‐MOF dosed with ^13^CO_2_ and C^17^O_2_, respectively. (C) Representative fit of the {^1^H}^17^O spectra (23.5 T, 2 0 kHz MAS, 1.2 h, one‐pulse) of the dosed KHCO_3_‐CD‐MOF with peak shapes generated from the DFT calculated values, taken from Figure 1, with apparent absence of hydroxyl environment (the right‐hand experimental peak is physisorbed CO_2_). (D) Comparison of the same {^1^H}^17^O spectra to KHCO_3_/K_2_CO_3_ ⋅ 1.5 H_2_O solid powders, natural abundance, (23.5 T, 20 kHz MAS, hahnecho. KHCO_3_ – 34.0 h, K_2_CO_3_ ⋅ 1.5 H_2_O – 42.1 h). See Figures S41‐S42 for PXRD patterns of solid crystal samples.

Initial ^13^C NMR experiments were performed on the ^13^CO_2_ dosed CD‐MOF, Figure 4A, to give context to the further ^17^O NMR experiments. Results in the chemisorbed CO_2_ region revealed two peaks clearly identifiable at 159 and 163 ppm. These are in agreement with the previously reported value of 159.6 ppm – only a single environment was previously assigned despite the apparent asymmetry of the chemisorbed CO_2_ peak.^[16]^ Consistent chemisorbed CO_2_ environments were reproduced for a range of samples (Figure S19). These values fall in the expected range for bicarbonate and carbonate anions (161–168 ppm in solution,^[80]^ and 160–169 ppm^[81]^ in solid‐potassium crystals), but it is hard to make conclusive assignments with any confidence.

The identification of two chemisorbed CO_2_ environments suggests either a mixed bicarbonate‐carbonate mechanism or a two‐site (bi)carbonate mechanism is likely occurring. Heats of adsorption data supports this with an ambiguous crossover between mechanisms (Figure S20). Cross‐polarisation (CP) MAS kinetic experiments were initially trialled, but remained inconclusive (Figure S21) and thus, we propose ^17^O NMR spectroscopy as having the potential to improve clarity of mechanistic assignments – particularly motivated by the direct impact the mechanistic difference between carbonate and bicarbonate has on material CO_2_ capacity.

The ^17^O NMR spectrum of the CD‐MOF dosed with C^17^O_2_ (Figure 4B), shows three peaks clearly resolved at *δ_obs_* =−12, 68 and 140–180 ppm. Physisorbed CO_2_ and chemisorbed CO_2_ are readily assignable at 68 and 140–180 ppm, respectively, in line with MFU‐4l and with previous work.^[29]^ The peak at negative ppm bares similarity to the Zn‐OH peak in MFU‐4l and is in a similar region to minor peaks previously assigned as framework defects in amine‐based MOFs.^[29]^ However, here we assign the peak as partially desolvated H_2_O within the MOF pore. This species will likely be in equilibrium with hydroxide, (bi)carbonate and CO_2_ (see equ (2‐3)) explaining both the ^17^O enrichment and the similarities (negative *δ_obs_*) to the Zn‐OH peak in MFU‐4l.

Further support for this H_2_O species assignment is found when comparing to the similarly partially solvated H_2_O in the DFT calculations in Figure 1, which place *δ*(H_2_^17^O)=−4 to −34 ppm. Additionally, as the pore environment is nominally ‘dry’ after MOF activation, any water present is expected to be in low concentration, and thus with a reduced hydrogen bonding network. Therefore, the observed peak position between that of fully solvated liquid water (0 ppm) and that of isolated gaseous water (−36.1 ppm),^[82]^ is consistent with that of a partially solvated species. Thirdly, the high hydrophilicity of the cyclodextrin ligand^[83]^ and the suggestion of previous binding energy calculations that the presence of H_2_O plays a key role in stabilising CO_2_ binding^[16]^ further supports the mechanistic involvement of H_2_O species.

To assign the chemisorbed CO_2_ environment a first comparison to the simple static DFT calculation was performed (Figure 4C), i. e. a slow exchange regime was assumed (see Figure 3A). This immediately gave the clear result that no static hydroxyl group is present in the CO_2_ binding mode in CD‐MOF. The calculated carbonyl environments are in broad agreement with the experimental spectrum giving two mechanistic possibilities: i) only carbonate species are present – i. e. no hydroxyl groups; ii) bicarbonate/hydroxyl groups are present but chemical exchange alters the spectrum.

The first of these possibilities is probed experimentally with comparison to solid powders of K_2_CO_3_ ⋅ 1.5 H_2_O and KHCO_3_, Figure 4D. The very good agreement found between the solid compounds and the CO_2_ dosed CD‐MOF indicates the presence of bicarbonate is still likely and, additionally, supports the possibility of a mixed carbonate‐bicarbonate mechanism. However, the absence of the hydroxyl environment in both the CD‐MOF and KHCO_3_(s) spectra indicate proton dynamics are significantly at play in both materials.

### Dynamic ^17^O NMR Modelling in KHCO_3_ and KHCO_3_‐CD‐MOF

**Figure 5:**
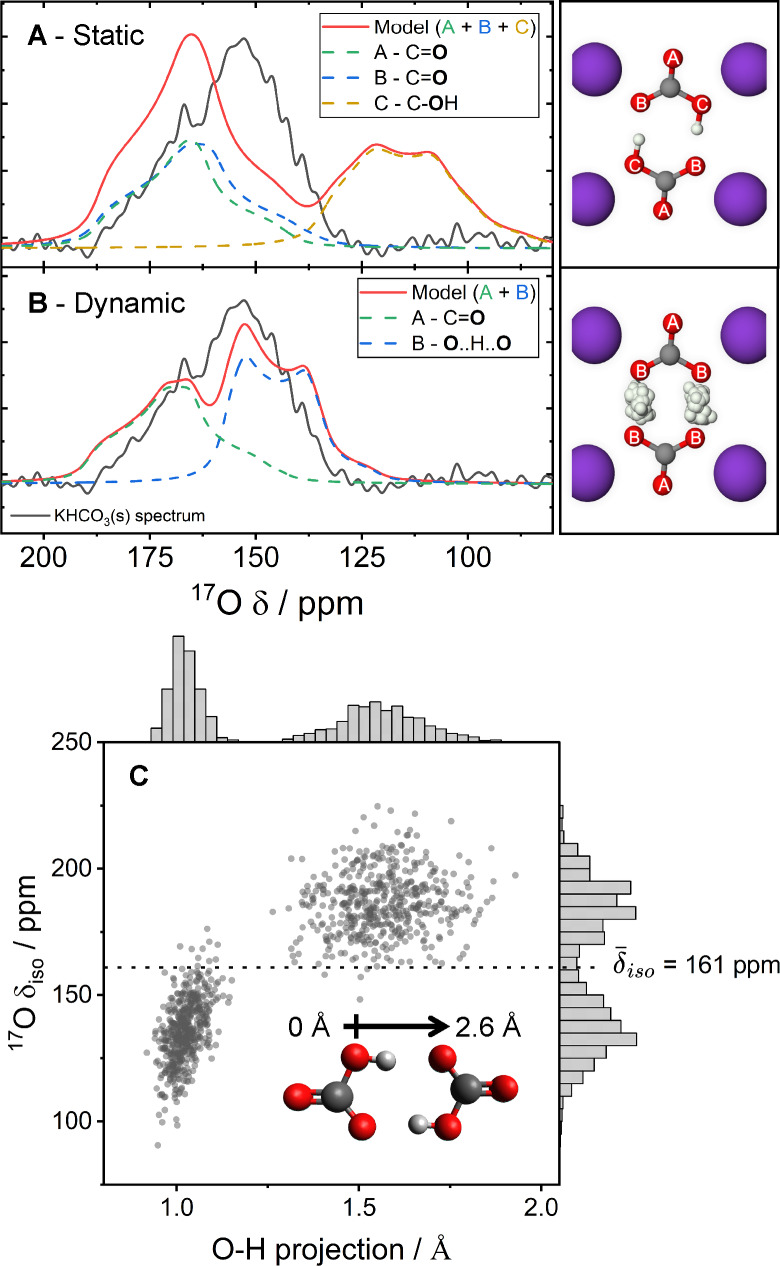
Fast regime dynamic modelling of KHCO_3_(s). Comparison of computational static (A) and dynamic (B) ^17^O NMR models compared to experimental spectra of KHCO_3_(s) (23.5 T, 20 kHz MAS, 33.0 h, hahnecho). To the side, illustrations of the crystal structure of KHCO_3_(s) show the unique oxygen (red) environments, as well as samples of the hydrogen (white) positions in the dynamic case (grey=carbon, purple=potassium). (**C**) A plot of ^17^O isotropic shift of all the now equivalent (under exchange) dynamic oxygen environments against the projection (onto the oxygen‐oxygen axis) of oxygen‐proton distance of all the MD samples, the mean *δ_iso_* is marked (further illustrations of the same data are shown in Figures S22–S23).

KHCO_3_(s) was investigated first through the modelling methods described in Figure 3. The bicarbonate dimer motif within the crystal structure, Figure 5A – right,^[84]^ is known to facilitate a rapid proton hop between the hydroxyl‐carbonyl pairs. The authors note a recent work provides differing NMR parameters for KHCO_3_ which we compare with our own results in Figure S25.^[89]^ However, in this work we train a MLFF to perform a transition state search and determine a proton hop frequency of 140 GHz (see Figure S24). This corresponds to the fast chemical exchange regime in NMR experiments (see Figure 3C).

MLFF‐MD simulations with NMR DFT sampling, Figure 5B–C, demonstrate clearly that rapid chemical exchange between the carbonyl oxygen – B, and hydroxyl oxygen – C, pairs gives a much improved two‐environment simulation (red) of the experimental spectrum (black). The symmetric proton hopping (Figure 5C) produces a fast exchange average value of *δ_iso_* =161 ppm, and, through electric field gradient (EFG) tensor averaging, a C_*Q*_=7.0 MHz and *η_Q_* =0.2 for this new ‘dynamic hydroxyl’ peak (see Table 1). (For clarity, the nomenclature of a ‘dynamic hydroxyl’ refers to an oxygen environment that is in fast exchange between a hydroxyl and carbonyl site). The parameters calculated here for the ‘dynamic hydroxyl’ match reasonably well with previously reported NQR results from *Poplett and Smith* of C_*Q*_=6.77–7.34 MHz and *η*_Q_=0.24–0.66. Additionally, small motions of the non‐exchanging carbonyl environment, A, leads to minor changes in parameters (see Table 2).

**Table 1:** Comparison of NMR parameters of the dynamic hydroxyl region of the experimental fits of the CO_2_ dosed CD‐MOF (see Figure 6A) and KHCO_3_(s) (Figure S26) and the MLFF‐MD calculated values for KHCO_3_(s) through cartesian tensor averaging.

	Experimental fits		MD averaged DFT
	CD‐MOF	KHCO_3_(s)	KHCO_3_(s)
*δ_iso_* / ppm	163	163	161
C_*Q*_ / MHz	6.4	6.1	7.0
*η_Q_*	0.6	0.6	0.2

**Table 2:** Comparison of NMR parameters of the carbonyl environments of the experimental fit of the CO_2_ dosed CD‐MOF (Figure 6A) and of the calculated carbonyl values of static DFT (cluster and CD‐MOF values, Figure 1) and of KHCO_3_(s)/K_2_CO_3_ ⋅ 1.5 H_2_O(s), both static and dynamic averaged (*KHCO_3_ is case C, fast exchange, K_2_CO_3_ ⋅ 1.5 H_2_O is case B, intermediate regime, see Figure 3).

	Expt. CD‐MOF fit		Cluster calculations		Static DFT		Dynamic average*	
	**1** – Blue	**2** – Purple	HCO_3_^−^	CO_3_^2−^	KHCO_3_(s)	K_2_CO_3_(s)	KHCO_3_(s)	K_2_CO_3_(s)
*δ_iso_* / ppm	192	181	174–209	174–193	188	206, 193, 184	190	191
C_*Q*_ / MHz	6.5	6.0	7.0–8.1	7.6–8.0	7.7	7.6, 7.5, 7.4	7.3	7.3
*η_Q_*	0.8	0.7	0.6–1	0.6–1	0.75	0.8, 1.0, 0.9	0.7	0.7

In a similar fashion, a refined fit for K_2_CO_3_(s) may be estimated through dynamic modelling. The three distinct carbonyl environments within the K_2_CO_3_ ⋅ 1.5 H_2_O structure are defined as having 0, 1 and 2 adjacent hydrogen bonds, respectively. Assuming an intermediate exchange regime (Figure 3B) of a C_3_ axis rotation, as previously seen in carbonate anion systems,^[91]^ *δ_iso_* and *η_Q_* values are produced with stochastic hop modelling using EXPRESS (see Figure S28). These are found to match reasonably to the experimental fit values (Tables 2 and S3),[^30^, ^91^, ^92^, ^93^] though the C_*Q*_ is slightly higher in the simulation.

**Figure 6:**
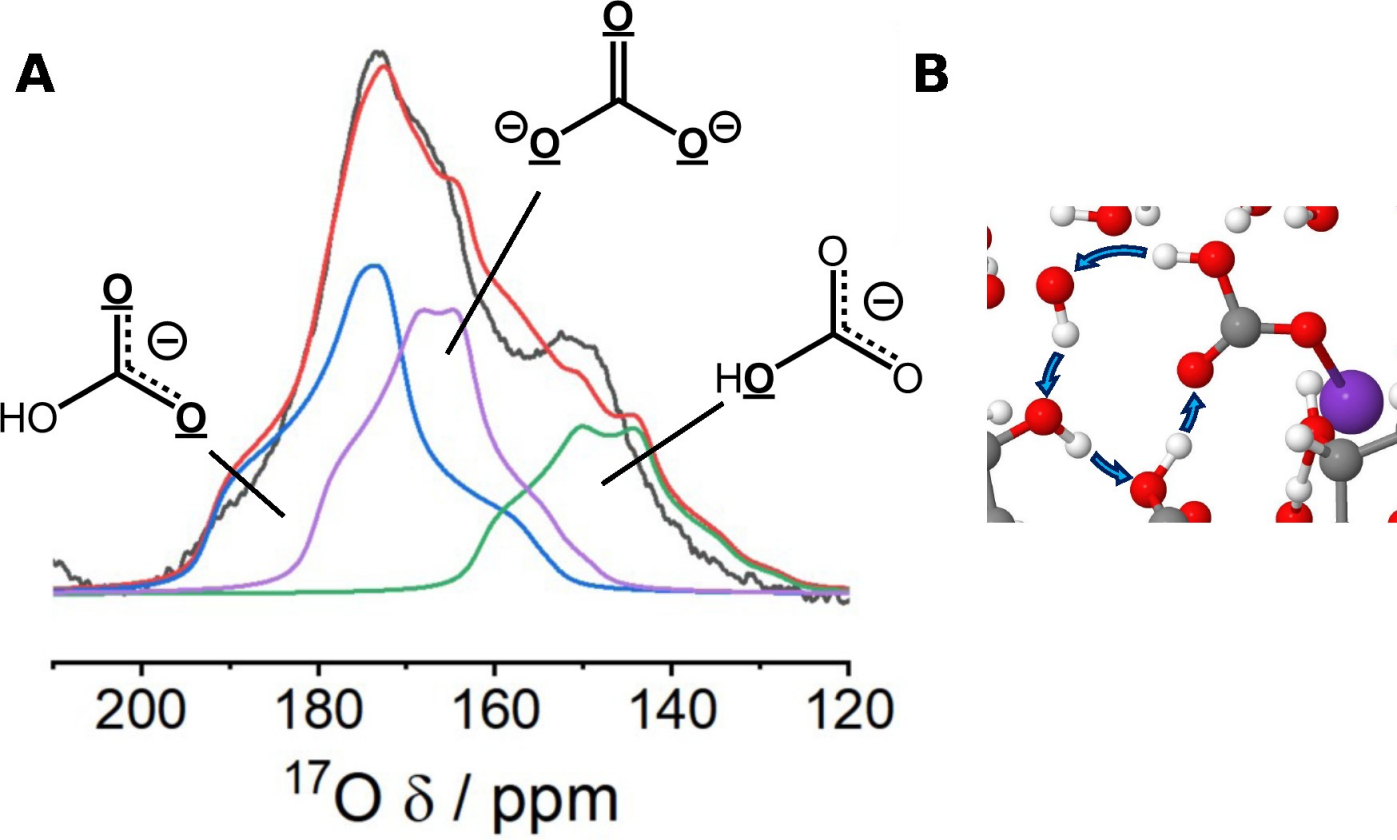
A new CO_2_ binding mechanism for KHCO_3_‐CD‐MOF. (**A**) An assigned {^1^H}^17^O NMR spectrum (23.5 T, 20 kHz MAS, 1.2 h, one‐pulse) of C^17^O_2_ dosed (0.8 bar) CD‐MOF with a three environment fit (blue, purple, green) derived from MQMAS and multi‐field fitting data (see SI). Black – experiment, red – summed model. (**B**) A snapshot from an MLFF‐MD simulation of the CD‐MOF demonstrating an example proton‐hopping pathway for the ‘dynamic hydroxyl’ environment.

The computational expense of DFT training and extensive DFT NMR calculations on the CD‐MOF system (>1000 atom unit cell) meant a combination of additional NMR experiments (Figure 6A), direct inference from the quantitative modelling of KHCO_3_ and K_2_CO_3_ (Tables 1 and 2), and qualitative MD simulations (Figure 6B)^[46]^ were utilised for mechanistic conclusions to be achieved. Experimentally, insight into the constituent environments of the chemisorbed CO_2_ peak was resolved via an MQMAS spectrum, supported by multi‐field fitting of ^17^O NMR spectra obtained at 20.0 and 23.5 T (see *MQMAS and multi‐field* ^*17*^ *O NMR fits* in the SI and Figures S30–S34 for further details). This identified three environments (Figure 6A) assigned as carbonyls and hydroxyls all with a large degree of broadening, symptomatic of dynamics being at play.

First, the lowest shift peak at *δ_iso_* =163 ppm is assigned as a ‘dynamic hydroxyl’ environment due to very good agreement to both the experimental and MLFF‐MD simulated values for KHCO_3_(s) (Table 1). Both the *δ_iso_* and C_*Q*_ values agree well, and *η_Q_* discrepancies are explained through broad lineshapes giving poorly defined line shape features for fitting. Additionally, in both of the more complex NMR experiments, MQMAS and double‐frequency sweep (DFS) (Figures S29‐S30), the expected signal enhancements of these techniques are reduced in the lower shift region of this ‘dynamic hydroxyl’, qualitatively supporting the idea that dynamics may be affecting signal accumulation.

The two higher shift peaks at *δ_iso_* =181, 192 ppm are more challenging to assign precisely, although both clearly fall in the carbonyl region from comparison to both calculated (Table 2) and experimental and literature results (Table S3).[^30^, ^92^, ^93^] From ^13^C NMR (Figure 4A), two carbon species are expected – at a ratio of ≈1:1.6 (Figure S35). Thus, the three oxygen environments assigned must correspond to two (bi)carbonate species of the same ratio. From the multi‐field data (Figure S34), the ratio of the 181 ppm peak to the combined total of the 163 and 192 ppm peaks remains constant at around 1 : 1.8–1.9. Given the broadness of the ^17^O NMR peaks, these ratios are in reasonable agreement with the ^13^C NMR. Thus, the 163 and 192 ppm ^17^O peaks can be tentatively assigned as belonging with the 159 ppm ^13^C peak as bicarbonate ‘dynamic hydroxyl’ and carbonyl environments, respectively. The 181 ppm ^17^O peak can thus be assigned with the higher shift ^13^C environment at 163 ppm as a carbonyl of a carbonate species. This is supported by the generally lower ^17^O *δ_iso_* values calculated for carbonate carbonyls compared to those in bicarbonates (Figure S6b, Table 2) – as well as generally higher ^13^C NMR shifts for carbonates (see Table S2).[^80^, ^81^]

The final piece of validation for the assignments in Figure 6 comes from MD simulations using the MACE‐MP‐0 pre‐trained ML force‐field.^[46]^ Recently introduced, MACE‐MP‐0 is widely applicable to a large variety of materials, in particular MOFs, without the need for additional and, in this case, expensive DFT training.^[46]^ Molecular dynamic simulations reveal the existence of extensive hydrogen bond network surrounding the active sites (see Figure S36). We investigate the plausibility of proton hops between hydroxyl, carbonyl, hydroxide and framework alcohol sites by assessing the thermodynamic stability of various hydrogen networks. We find that a carbonyl‐hydroxyl proton transfer can be facilitated by a proton hop along the hydrogen network (Figures 6B and S36), providing a plausible mechanism for a dynamic hydroxyl group within the bicarbonate. Additionally, the similarity in hydrogen bonding environment of the (bi)carbonate anions in the CD‐MOF system to other encapsulated anion systems where dynamics are well established^[91]^ further supports the likelihood of general dynamics (e. g. rotational/translational) in addition to exchanging proton sites.

These assignments give the new mechanistic result that KHCO_3_‐CD‐MOF has a mixed carbonate‐bicarbonate mechanism at an approximate 1:1.6–1.9 ratio (at 0.8–1.1 bar CO_2_). This explains the ambiguous heats of adsorption data^[16]^ (Figure S20) which sees a chemisorption‐physisorption switch matching well to hydroxide saturation at a suggested 1:1.6–2.4 ratio of carbonate:bicarbonate.

In order to improve clarity of assignments and provide a more complete picture of dynamic behaviour, variable temperature NMR experiments were attempted. However, the complex system within the NMR rotor for CO_2_ capture materials – including chemisorbed, physisorbed and free head‐space gaseous CO_2_ – presents a multi‐component equilibrium system that is itself affected by temperature changes. This resulted (Figure S37) in alterations of the NMR spectrum that are not simply due to dynamic effects but also driven by shifts in equilibrium position with temperature (e. g. increased CO_2_(g) adsorption with decreasing temperature). This experimental limitation further justified MLFF‐MD modelling as a crucial tool for providing insight into the effects of dynamics in the NMR spectroscopy of CO_2_ capture systems.

## Conclusions

This work has demonstrated the significant power and considerations required for applying ^17^O NMR spectroscopy to the study of hydroxide‐based CO_2_ capture materials. Initial static DFT results suggested ^17^O NMR should provide clear diagnostic assignments of hydroxyl, carbonyl and water environments with respect to their *δ_iso_* and C_*Q*_ values. Experimental results on MFU‐4l and KHCO_3_‐CD‐MOF corroborated these results to an extent, with chemisorbed and physisorbed CO_2_ and water/hydroxide readily assignable from 1D ^17^O NMR spectra. However, more sophisticated MLFF‐MD simulations were demonstrated as efficient and effective models for the influences of both carbonyl and hydroxyl dynamics in the spectra of the two MOFs.

For MFU‐4l, our results supported a previously proposed bicarbonate capture mode, and a two aspect fast‐intermediate exchange regime was identified involving carbonyl ‘seesaw’ and proton swivelling motions. KHCO_3_‐CD‐MOF required a multi‐faceted analysis approach, including: experimental ^17^O NMR comparison to model solid compounds KHCO_3_ and K_2_CO_3_ ⋅ 1.5 H_2_O with quantitative MLFF‐MD modelling, 2D MQMAS and multi‐field data, and qualitative MLFF simulations. These combined to give a newly proposed mixed carbonate‐bicarbonate mechanism, 1:1.6–1.9 ratio, for KHCO_3_‐CD‐MOF. Overall, this work shows the potential of ^17^O NMR spectroscopy to reveal carbon dioxide speciation and dynamics, and thereby to act as a guide for the design and development of improved hydroxide‐based CO_2_ capture materials.

## Conflict of Interests

The authors declare there are no conflicting interests.

1

## Supporting information

As a service to our authors and readers, this journal provides supporting information supplied by the authors. Such materials are peer reviewed and may be re‐organized for online delivery, but are not copy‐edited or typeset. Technical support issues arising from supporting information (other than missing files) should be addressed to the authors.

Supporting Information

## Data Availability

The NMR spectra, diffraction, computational and structural data generated in this study have been deposited in the Cambridge Research Repository, Apollo, at DOI reference: https://doi.org/10.17863/CAM.109966.
