# Supplementary material for: 17O NMR Spectroscopy Reveals CO2 Speciation and Dynamics in Hydroxide‐Based Carbon Capture Materials
Source: Chemphyschem. 2024 Dec 16;26(5):e202400941. doi: 10.1002/cphc.202400941 (PMC11878753; doi:10.1002/cphc.202400941)
Supplement: Supplementary file 1 — Supporting Information [file CPHC-26-e202400941-s001.pdf]

ChemPhysChem

Supporting Information

# $^{17}O$ NMR Spectroscopy Reveals $CO_2$ Speciation and Dynamics in Hydroxide-Based Carbon Capture Materials

Benjamin J. Rhodes, Lars L. Schaaf, Mary E. Zick, Suzi M. Pugh, Jordon S. Hilliard, Shivani Sharma, Casey R. Wade, Phillip J. Milner, Gábor Csányi, and Alexander C. Forse*

# Supporting Information

## Contents

**Experimental section** 1
Materials 1
PXRD and gas sorption 2
Static DFT methods 2
Modelling dynamics with machine-learning force fields 3
NMR Spectroscopy 3
Dosing of enriched $^{13}CO_2/C^{17}O_2$ gas 4
NMR acquisition parameters 4

**Additional Figures** 5
DFT clusters, structures and additional calculation results 5
Further NMR and MLFF molecular dynamic simulations 11
MQMAS and multi-field $^{17}O$ NMR fits 20
Additional NMR, quantification and CD-MOF MLFF structures 23
Materials characterisation 27

## Experimental section

### Materials

All of the chemicals used in this project were purchased from commercial suppliers and were used without further purification. $^{13}C$-enriched $CO_2$ gas was purchased from Sigma-Aldrich with 99.0 atom % $^{13}C$, $^{17}O$-enriched $CO_2$ gas was purchased from ICON/Berry & Associates Inc., with ∼20 atom % $^{17}O$.

#### $KHCO_3$(s) & $K_2CO_3$(s)

The samples of $KHCO_3$ and $K_2CO_3$ were both obtained from ThermoFischer Scientific Inc.. $K_2CO_3$ was quoted as anhydrous, 99+% purity. PXRD patterns of the same chemical batch as used for NMR spectroscopy are shown in Figs. S41-S42. These revealed that the $K_2CO_3$ was in fact in the $1.5\,H_2O$ hydrated form, likely as a result of prolonged air exposure.

#### MFU-4l MOF

MFU-4l MOF was synthesised following the TBAOH exchange method as described in Reference [1].[1] The MOF was characterised by PXRD (Fig. S43) and $N_2$ and $CO_2$ isotherms (Fig. S44). The PXRD were performed on the $CO_2$ loaded form of the MOF and for the isotherm and all NMR measurements the MOF sample was pre-activated at 100°C, vacuum for at least 12 h. MOF samples were handled in a $N_2$ glovebox (incl. for NMR rotor packing) to avoid moisture and atmospheric $CO_2$ exposure.

#### $KHCO_3$-CD-MOF

$KHCO_3$-CD-MOF synthesis was performed as previously reported in the literature.[2] $\gamma$-cyclodextrin (324 mg, 0.25 mmol, 1.00 eq.), potassium bicarbonate (200 mg, 2.00 mmol, 8.00 eq.), methanol (8 ml), and deionized water (2 ml) were combined in a 25 ml Teflon autoclave. The mixture was sonicated and stirred at room temperature for 1 h. The autoclave was sealed and heated in an oven (140 °C, 4 days). The reaction was slowly cooled to room temperature in the oven following heating. The brown crystals were transferred and soaked in solvent (3 × 3 ml methanol then 3 × 3 ml dichloromethane), each soak was for 24 h before decanting and replacement with

fresh solvent. The crystalline product was filtered and activated under flowing $N_2$ (RT, 24 h) then under vacuum (RT, 24 h). Activated $KHCO_3$ CD-MOF was obtained as orange-brown crystals.

Some challenges were found in reproducing material quality as assessed by characterisation via PXRD (see Fig S45) and gas sorption $N_2$ and $CO_2$ isotherms (see Figs. S46-S47). Despite this material variability, NMR results proved consistent between different samples synthesised by different researchers (Fig. S19 and S48. See Table S4 for specific sample information). The highest quality sample (sample 3) was utilised for obtaining the MQMAS and high-resolution 23.5 T NMR data. MOF samples were handled either in a $N_2$ glovebox/glovebag (incl. for NMR rotor packing) to minimise moisture and atmospheric $CO_2$ exposure.

## PXRD and gas sorption

Power X-ray diffraction (PXRD) data were obtained using a Malvern Panalytical Empyrean and Xpert instrument with non-monochromated CuK$\alpha$ radiation ($\lambda$ = 1.5406, 1.5444 ) and an X'celerator Scientific detector. Measurements were performed at room temperature on 5 mm or 10 mm sample area glass disks. Divergence and anti-scatter slits of 1/8" and 1/4", respectively, were use to achieve $2\theta$ ranges of 3-50° or 5-40° with step size = 0.017°.

All gas adsorption isotherms, apart from Fig. S44, for $CO_2$ and $N_2$ were taken on an Anton Parr Autosorb iQ-XR analyser. Samples were activated on the attached vacuum degassing stations ($KHCO_3$ CD-MOF room temperature = 303 or 308 K, 24 h). $N_2$ isotherms were obtained at 77 K (liquid $N_2$ bath), and $CO_2$ isotherms were taken at either 298 K or 303 K (water bath). All analysis was performed in AsiQwin 5.21 software, where multipoint BET was applied to $N_2$ isotherms for surface area determination.

Fig. S44 was taken on a different adsorption instrument using a Micromeritics 3Flex Surface Characterization Analyzer. These measurements were performed using ultra-high-purity-grade gases (99.999%) purchased from Praxair ($N_2$, NI 5.0UH-K; $CO_2$, CD 5.0LS-K). Prior to analysis, samples (100–200 mg) were transferred to oven-dried and tared sample tubes equipped with TranSeals (Micrometrics) and heated to 100 (1 °C min$^{-1}$) under vacuum until the outgas rate was less than 0.0033 mbar min$^{-1}$. The $N_2$ isotherm was collected first at 77 K, followed by a degassing step at 100 °C for 6 h to remove any physisorbed $N_2$ prior to $CO_2$ adsorption analysis at 300 K. A Micrometrics thermocouple-controlled heating mantle was used to maintain the sample temperature.

## Static DFT methods

The hydration cluster calculations were performed using Gaussian16 [3] using the B3LYP functional and the aug-pcSseg-2 [4] and 6-311+G(d,p) basis sets. Starting structures were generated in Avogadro [5] in line with clusters previously presented in the literature. [6–9] Structures were initially optimised with the 6-311+G(d,p) basis set and NMR parameters calculated. The optimised structures were then reoptimised in the more computationally expensive (and more accurate with respect to NMR chemical shielding) aug-pcSseg-2 basis set. [4] Structures that were below 2.6 kJmol$^{-1}$ (= kT at T = 308 K, as chosen to be representative of room temperature plus an approximated 10°C of MAS frictional heating) in energy relative to the ground-state (in either basis-set) were included in NMR parameter maps (Fig. 1). A full list of the cluster structures considered with their respective energies are included in Figs. S1-S2.

The $^{17}$O chemical shielding reference value for these cluster calculations was taken from a linear fit ($\delta_{iso} = \sigma_{iso} - \sigma_{ref}$) of a range of experimental $^{17}$O $\delta_{iso}$ values with fixed gradient $-1$. The experimental values were taken from $CO_2$, CO, $H_2O$, $N_2O$, OCS, $OF_2$, $H_2O_2$, $H_2O_3$ and $Et_2O_2$ giving $\sigma_{ref}$ = 280 for aug-pcsSeg-2 and $\sigma_{ref}$ = 284 for 6-311+G(d,p) (see Fig. S40). [10,11]

Periodic plane-wave DFT calculations were performed in CASTEP [12] using the default on-the-fly pseudopotentials and Perdew-Burke-Ernzerhol (PBE) functional with a G06 van der Waals correction. The magnetic shielding tensors and electric field gradient tensors were computed in CASTEP, which uses density functional perturbation theory and the GIPAW method [13–15].

The periodic MFU-4l structure was generated from the crystal structure of MFU-4l-Cl from *Denysenko et al.*. [16] This unit cell was reduced to a rhombohedral unit cell ($a$ = 21.961 Å, $\alpha$ = 60°) of 162 atoms. The chlorine atoms were replaced by -OH groups followed by geometry optimisation. Subsequently, a singular -OH group was replaced by varied -$HCO_3$ starting binding configurations from which the two mode-A and mode-B (Fig. S10) structures where derived through further geometry optimisation, in agreement with previous literature cluster calculations. [1] Geometry optimisation tolerances used were: force tolerance = 0.05 and 0.075 eVÅ$^{-1}$, displacement tolerance = 0.002 and 0.003 Å for the -OH and -$HCO_3$ modes, respectively, and an energy tolerance = $2 \times 10^{-5}$ eV atom$^{-1}$ was used for both.

For the four crystal structures considered: MFU-4l, $KHCO_3$(s), [17] $K_2CO_3 \cdot 1.5\ H_2O$(s) [18] and $KHCO_3$-CD-MOF, [2] individual convergence for NMR parameter calculations of cut-off energy ($E_{cut}$) and Monkhorst-Pack k-point grid spacing were performed. For MFU-4l (both $CO_2$ binding modes used the same parameters), an $E_{cut}$

= 650 eV and k-point spacing = 0.04 Å$^{-1}$ (grid = 2 2 2) were used giving convergence within $\sigma_{iso}$ = 0.5 ppm, $C_Q$ = 0.01 MHz and $\eta_Q$ = 0.002 (the values given are root-mean-squared average parameter difference, across all oxygen atoms, between the chosen $E_{cut}$/k-point spacing and the subsequent higher accuracy cut-off/spacing of the convergence test. The largest average deviation of the the two control variables is quoted). For the MLFF molecular dynamics NMR sampling of MFU-4l was performed without dispersion correction. For $KHCO_3$(s) an $E_{cut}$ = 1400 eV and k-point spacing = 0.02 Å$^{-1}$ (grid = 14 9 4) gave convergence within $\sigma_{iso}$ = 0.03 ppm, $C_Q$ = 0.001 MHz and $\eta_Q$ = 0.00. $K_2CO_3$·1.5 $H_2O$(s) had an $E_{cut}$ = 1100 eV and k-point spacing = 0.04 Å$^{-1}$ (grid = 3 2 5) with convergence within $\sigma_{iso}$ = 0.01 ppm, $C_Q$ = 0.001 MHz and $\eta_Q$ = 0.00. $KHCO_3$-CD-MOF was found more computationally expensive to converge due to its large unit cell size of 1050 atoms, however, convergence was found at $E_{cut}$ = 1769 eV and k-point grid = 1 1 1, with convergence within $\sigma_{iso}$ = 0.02 ppm, $C_Q$ = 0.003 MHz and $\eta_Q$ = 0.00.

Atomic positions were reoptimised with the converged $E_{cut}$ and k-point grid values and the NMR parameters subsequently calculated. A $^{17}$O $\sigma_{ref}$ value was obtained from a linear fit from experimental values of tyrosine and valine[19] and calculated values. For each amino-acid a (converged) k-point spacing of 0.04 Å$^{-1}$ was used along with the respective $E_{cut}$ for each crystal considered. All $E_{cut}$ values with dispersion correction gave $\sigma_{ref}$ value = 247 ppm and without dispersion correction = 250 ppm to 3 significant figures.

## Modelling dynamics with machine-learning force fields

We use MACE[20], a many-body equivariant graph neural network, that obtains state of the art performance on established benchmark datasets and extrapolation tasks[21]. The training set was curated using an active learning protocol outlined in Reference [22][22]. New configurations are sampled from MD simulations, when the predicted relative force uncertainty of any atom, obtained from the discrepancy among five committee models, exceeds 50%. In this manner new configurations are iteratively added to the training set.The MLFF for $KHCO_3$ is trained from scratch, while the MFU-4l MLFF is fine-tuned from the MACE-MP-0 foundation model[23]. During fine-tuning all weights in the model are allowed to change.

For the MFU-4l dynamics model, the NMR parameters are obtained by performing 18 NMR-DFT calculations on samples taken at 100 ps intervals from a 2 ns MD simulation, following a 200ps warmup, run with 0.5 fs time steps. An animation of a section of the trajectory is included in the supporting information. For the $KHCO_3$(s) model, the NMR parameters are obtained by performing 120 NMR-DFT calculations on samples from a 900 ps MD simulation, run with 0.5 fs time steps.

When predicting rates for the intermediate regime, we use the climbing-image nudged elastic band method to find the transition states. Again using active learning as in Reference [22][22] to improve the MLFF. We then do a frequency analysis to obtain reaction rates under the harmonic transition state theory. All simulations of the CD-MOF were done directly with the MACE-MP-0 foundation model, without further finetuning.[23] The simulation analysed included 1 ns of MD simulation at 0.5 fs time steps.

Intermediate exchange rates were modelled using EXPRESS.[24] Input NMR parameters were taken from DFT/MD results. The relative chemical shift anisotropy (CSA) and EFG Euler angles were obtained using MagresView[25] where one angle set was arbitrarily defined as [0 0 0] with powder averaging providing iteration over all orientations. For the 'fast-intermediate' case, the MD averaged tensor was used as an input in MagresView. The rate constants used for each EXPRESS simulation were: MFU-4l - 2.4 x $10^5$ Hz, $KHCO_3$(s) 1.4 x $10^{14}$ Hz and $K_2CO_3$.1.5$H_2O$(s) 1 x $10^4$ Hz.

## NMR Spectroscopy

Three NMR spectrometers were used to collect the data in this report. The $^{13}$C NMR data were collected on a 9.4 T wide bore magnet with a Bruker Avance NEO spectrometer using a 4 mm double resonance probe. $^{17}$O NMR experiments were recorded at the UK High-Field Solid-State NMR Facility on a 20.0 T Bruker Avance NEO spectrometer with a low-$\gamma$ 4 mm double resonance probe or on a 23.5 T Bruker Avance NEO, narrow bore, with a CP MAS double resonance DVT 3.2 mm probe. 4 mm rotors were spun at 12.5 kHz MAS (at both high and low field) and 3.2 mm rotors were spun at 20 kHz. Chemical shift references of $^{13}$C - adamantane = 29.5 ppm (−CH, right-hand peak) and $^{17}$O - liquid $H_2O$ = 0 ppm were used.

For each experiment, pulse lengths were individually optimised on a reference material ($^{13}$C - adamantane, $^{17}$O - $H_2O$(l)). For the $^{17}$O quadrupolar $\pi/2$-pulse on the solid samples themselves, the low RF limit ($\omega_Q > \omega_{rf}$)[26] was invoked such that the $\pi/2$-pulse utilised was derived from $3 \times \omega_{rf}$ measured on liquid $H_2O$. Full experimental parameters for all spectra in this work are listed in Table S1.

For the MQMAS experiment (Fig. S30), the z-filter pulse sequence was used to acquire the spectrum. The experimental parameters used were as follows: recycle delay (d1) = 0.02 s, 121,752 transients, 44 h, 3Q-conversion

pulses $\omega_1 = 66.7$ kHz, z-filter $\omega_2 = 20.8$ kHz. 32 rows were collected in the F2 dimension. Shearing was performed using the TopSpin *xfshear* function.[27]

NMR analysis was performed in Topspin v4.1.4 and spectral fitting was performed in dmFit[28] and ssNake.[29]

The quadrupolar parameter definitions used throughout this work are as follows (where the convention for PAS components utilised is $|V_{zz}| > |V_{yy}| > |V_{xx}|$):

$$C_Q = \frac{eQV_{zz}}{h}, \tag{1}$$

$$\eta_Q = \frac{V_{xx} - V_{yy}}{V_{zz}}. \tag{2}$$

$e$ is the fundamental electron charge and $Q$ is the nuclear electric quadrupolar moment.

It is noted that for the MD averaging, the EFG tensor of each individual MD frames are averaged in the same fixed Cartesian frame before subsequent diagonalisation to obtain the averaged PAS components (and thus quadrupolar parameters).[30–34]

## Dosing of enriched $^{13}CO_2/C^{17}O_2$ gas

The dosing of enriched $CO_2$ on MFU-4l and CD-MOF samples was performed using the same procedure and setup for *ex*-situ rotor dosing previously described, using 3.2 mm or 4 mm MAS rotors.[35,36] In the procedure, the MAS rotor is packed and then placed onto a custom-built vacuum/dosing line with the rotor cap resting (but not inserted) at the mouth of the rotor. The sample and gas line were evacuated for 10-15 mins, then enriched $CO_2$ gas is released into the evacuated line to dose the sample. Sample dosing pressures were between 0.7-1.3 bar with dosing performed for 10-30 mins to allow for gas adsorption equilibration, i.e. until no further pressure drop was observed. A plunger is then used to secure the cap and seal the rotor under the pure $CO_2$ environment, before removing to perform NMR measurements.

## NMR acquisition parameters

**Table S1.** A tabulation of the experimental acquisition parameters for all NMR experiments presented in both the main text and the following supplementary figures. Where further parameters are significant for the more complex sequences, details are provided alongside the spectrum in the figure caption.

| Figure | Number of transients | Recycle delay / s | $\omega_{rf}$ / kHz | Time / hr |
|---|---|---|---|---|
| Fig. 2C, 3, S18 | 288,784 | 0.1 | 43.9 | 10.8 |
| Fig. 4A | 1024 | 6 | - | 1.7 |
| Fig. 4B | 440,560 | 0.1 | 64.1 | 16.2 |
| Fig. 4C, 6A, S33, S34a | 32,768 | 0.1 | 66.7 | 1.2 |
| Fig. 4D, 5A/B, S26(a) | 237,536 | 0.5 | 52.1 | 34.0 |
| Fig. 4D, S27(a), S28(b) | 284,672 | 0.5 | 55.6 | 42.1 |
| Fig. S12 | 61,440 | 0.1 | 47.1 | 2.6 |
| Fig. S17 | 2048/102400 | 0.1 | 49.0 | 0.1/4.7 |
| Fig. S19 | 512-1504 | 6-14 | - | - |
| Fig. S21 | 1504 | 6 | - | 2.5 |
| Fig. S30 | 121,752 x 32 | 0.02 | 66.7 and 20.8 | 44.3 |
| Fig. S34b | 17,408 | 1 | 55.6 | 4.8 |
| Fig. S35 | 192 | 300 | 43.5 | 16.0 |
| Fig. S26(b) | 150,000 | 0.5 | 58.2 | 23.3 |
| Fig. S27(b) | 100,368 | 0.5 | 58.2 | 15.6 |
| Fig. S37 | 17,408 | 0.1 | 55.6 | 0.8 |

# Additional Figures

Figures complementary to the main text are included below in order in which they are referred to in the main text.

## DFT clusters, structures and additional calculation results

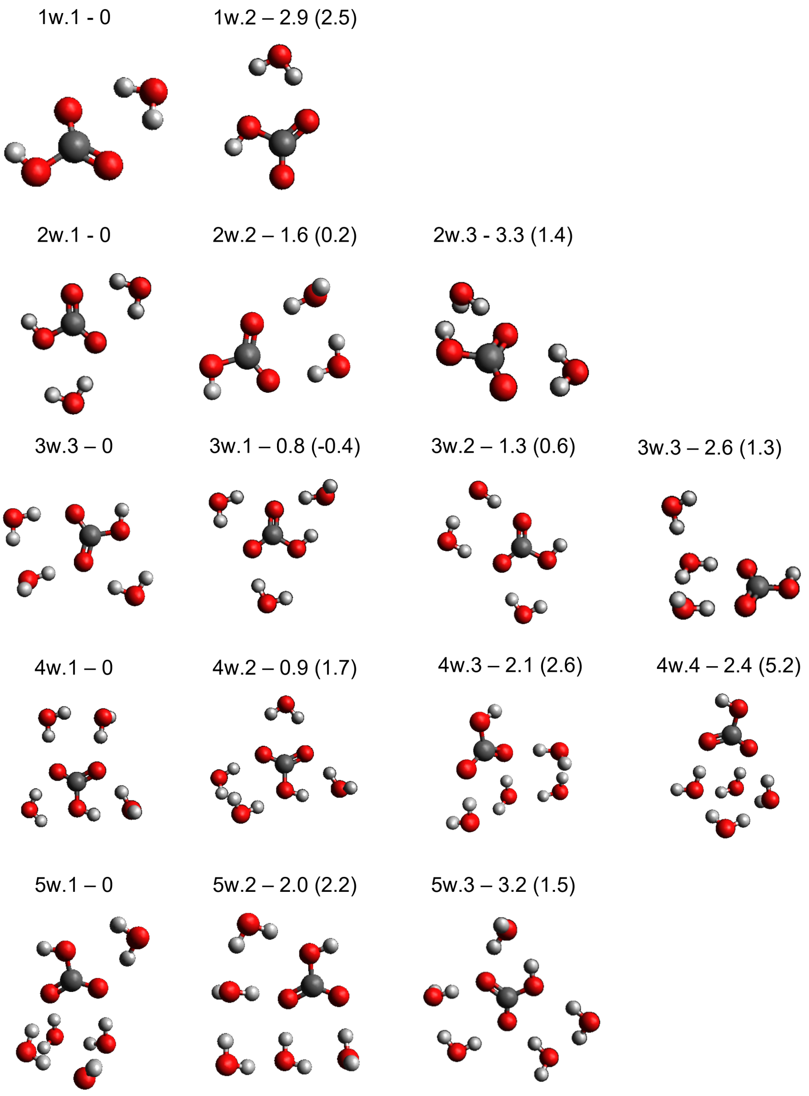


**Figure S1.** Structures used for the hydrated bicarbonate anion clusters used to calculate NMR parameters given in Fig. 1. Energies for each cluster are given in kJ mol$^{-1}$ relative to the ground state configuration, as calculated using the 6-311+G(d,p) basis set (aug-pcSseg-2 values are given in brackets). Structures are in agreement with the literature, with variations in energy ordering seen with basis set and functional choice, with relative energy fluctuation to literature of $\pm 2 - 3$ kJ/mol.[6–9]

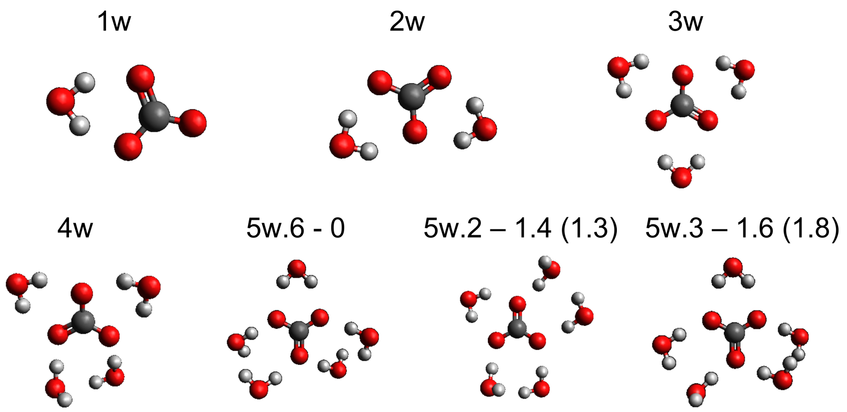


**Figure S2.** Structures used for the hydrated bicarbonate anion clusters used to calculate NMR parameters given in Fig. 1. For 5w, energies for each cluster are given in kJ mol$^{-1}$ relative to the ground state configuration, as calculated using the 6-311+G(d,p) basis set (aug-pcSseg-2 values are given in brackets). For 1-4w, no additional structures were found within 2.6 kJ mol$^{-1}$ of the ground state. Structures for 1-4w agree with literature [6,9], however, for 5w a variety of structures were found to contrast to the ground state published in Reference [9] [9] with structure search unable to find the proposed minimum. This discrepancy is attributed to differing calculation methodologies.

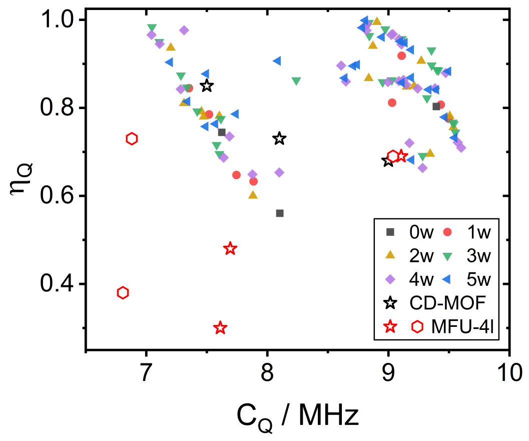


**(a)**

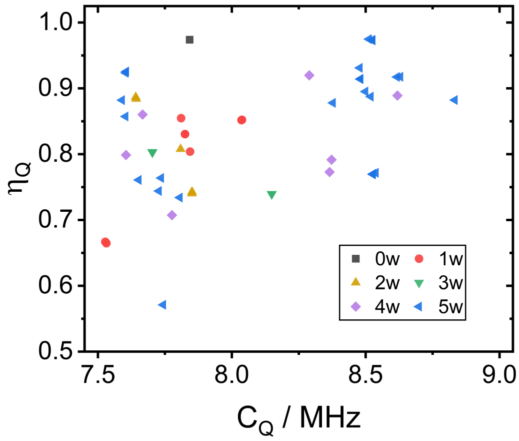


**(b)**

**Figure S3.** Plots of $\eta_Q$ vs $C_Q$ for the same cluster calculations presented in Fig. 1 for **(a)** bicarbonate and **(b)** carbonate hydrated ion clusters and MOF structures.

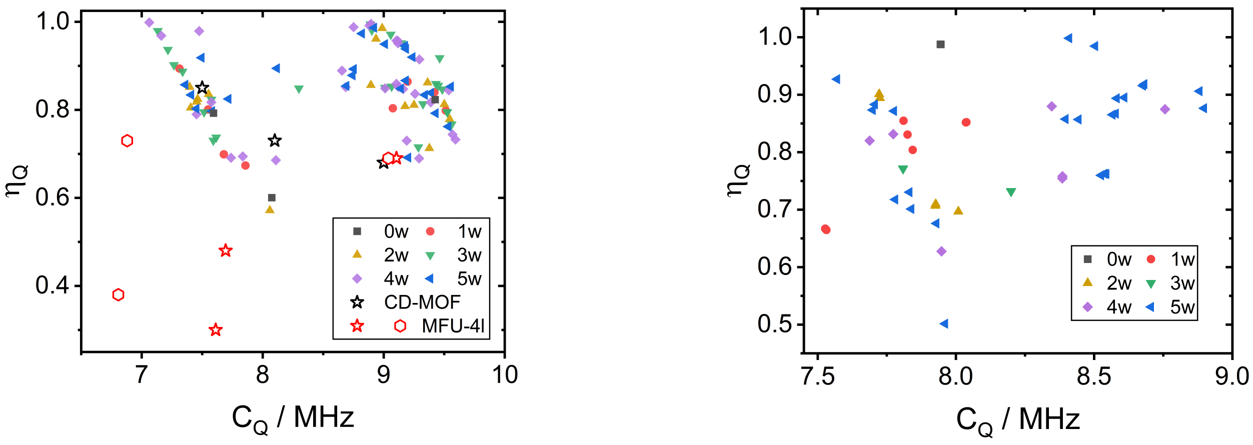


**(a)** **(b)**

**Figure S4.** Analogous plots of $\eta_Q$ vs $C_Q$ as Fig. S3 calculated on the same bicarbonate **(a)** and carbonate **(b)** clusters with a 6-311+G(d,p) basis set. Plane-wave basis set calculations of MFU-4l and $KHCO_3$-CD-MOF are also included in **(a)**.

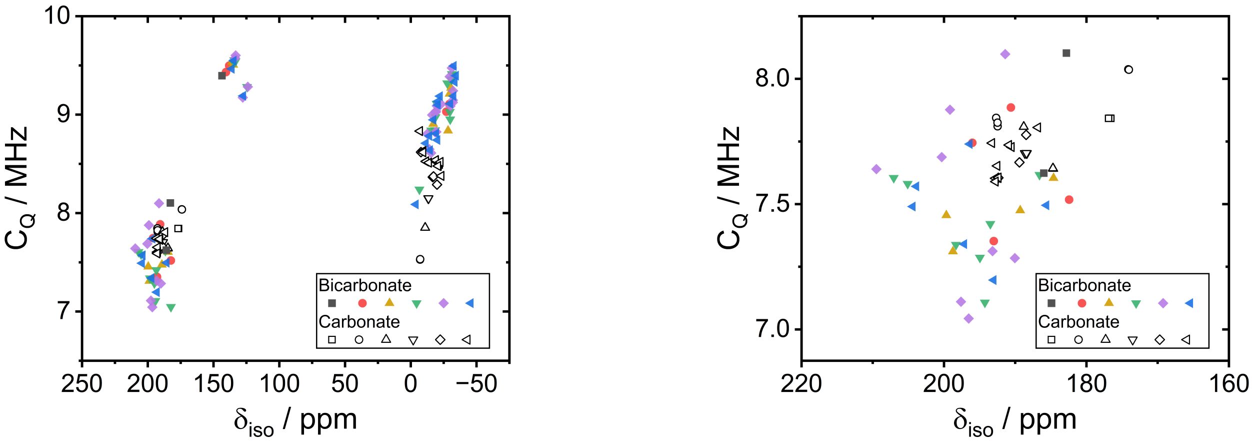


**(a)** **(b)**

**Figure S5.** Overlays of the bicarbonate and carbonate cluster calculations features in Fig. 1 at B3LYP/aug-pcSseg-2 level of theory. **(b)** shows a enlarged region of **(a)** in the carbonyl region.

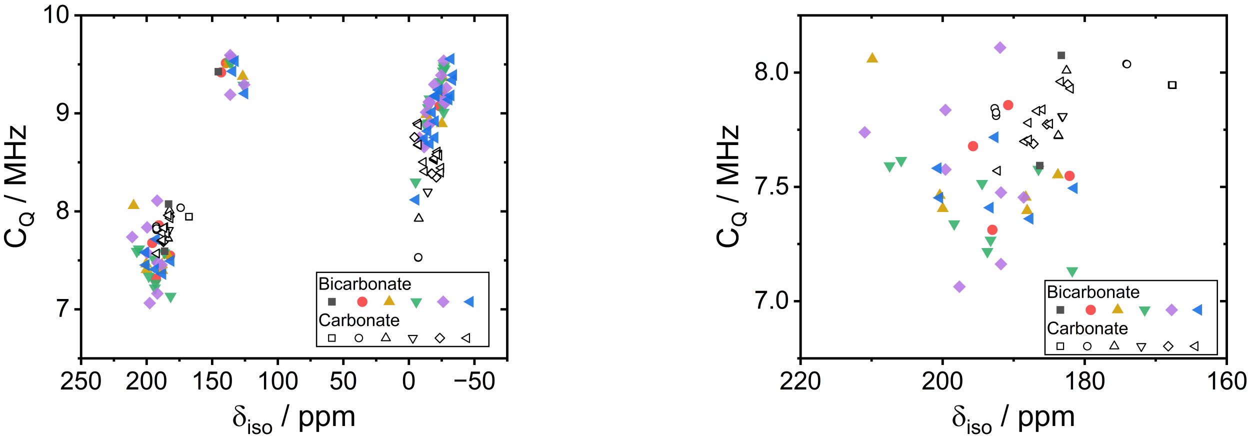


**(a)** **(b)**

**Figure S6.** Overlays of the bicarbonate and carbonate cluster calculations features in Fig. 1 at B3LYP/6-311+G(d,p) level of theory. **(b)** shows a enlarged region of **(a)** in the carbonyl region.

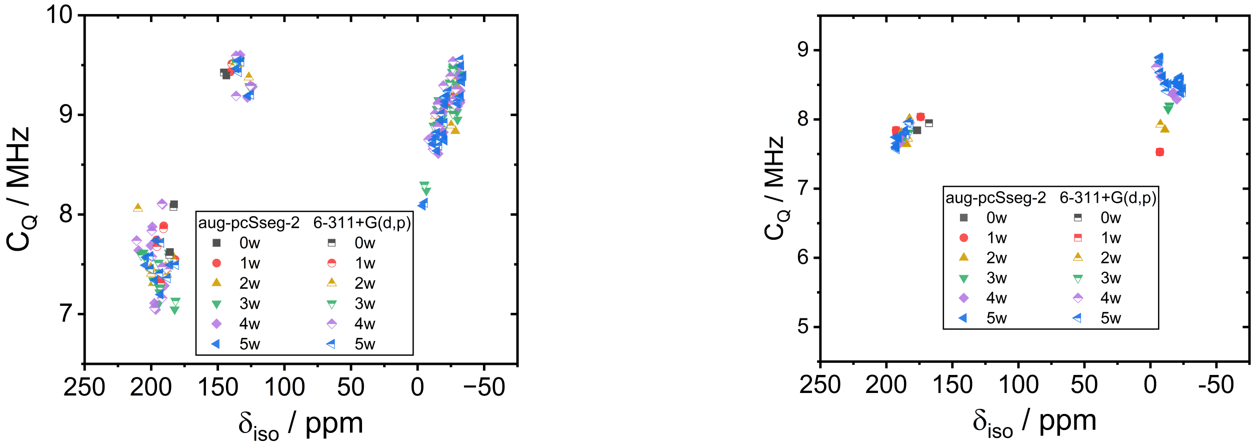


**(a)** **(b)**

**Figure S7.** Comparison of two basis-sets aug-pcSseg-2 and 6-311+G(d,p) for **(a)** bicarbonate and **(b)** carbonate clusters. aug-pcSseg-2 data reproduced from Fig. 1.

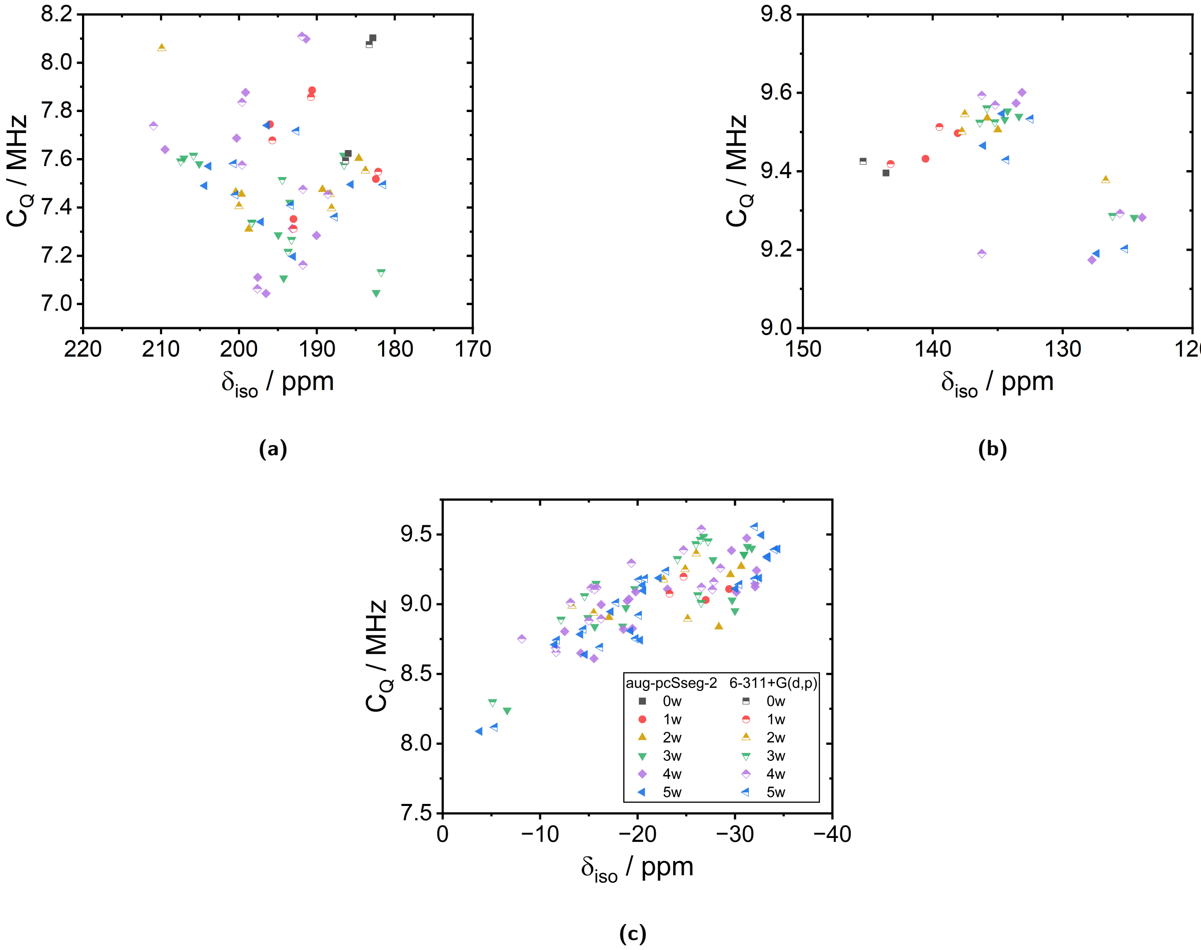


**Figure S8.** Zoomed section of Fig. S7a for **(a)** the carbonyl region, **(b)** the hydroxyl region and **(c)** the $H_2O$ region for comparison of the two basis-sets aug-pcSseg-2 and 6-311+G(d,p) in bicarbonate clusters.

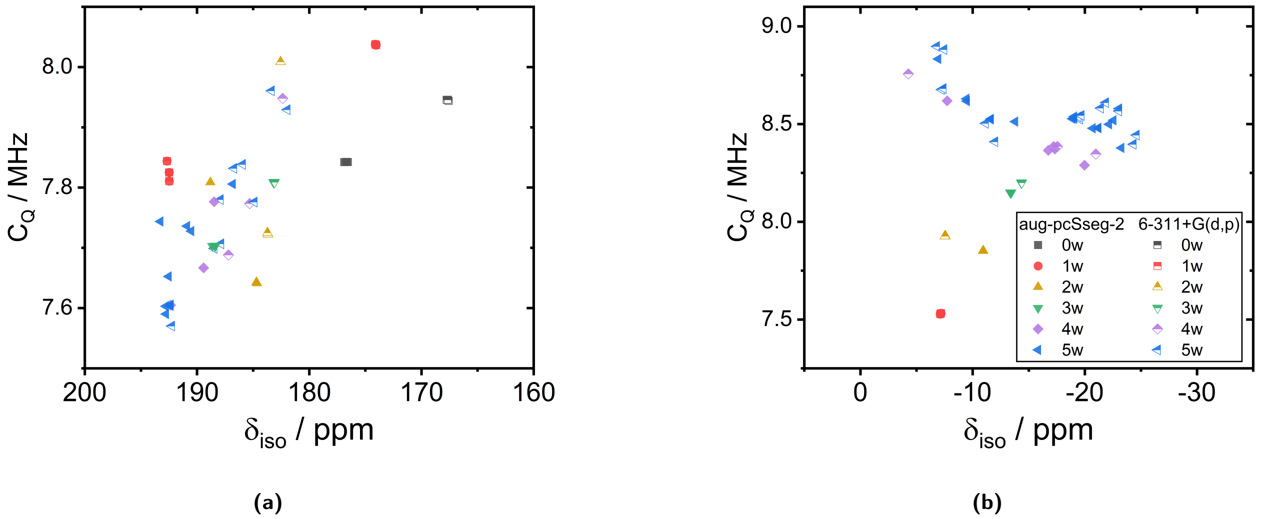


**Figure S9.** Zoomed section of Fig. S7b for **(a)** the carbonyl region and **(b)** the $H_2O$ region for comparison of the two basis-sets aug-pcSseg-2 and 6-311+G(d,p) in carbonate clusters.

**Table S2.** Average $^{13}$C NMR $\delta_{iso}$ values for the hydrated bicarbonate and carbonate cluster models presented above and in Fig. 1 (0w, non-hydrated structures excluded). $\sigma_{ref}$ values of 182.3 and 179.6 ppm are used for the 6-311+G(d,p) and aug-pcSseg-2 results, respectively, as obtained from reference calculations on a singular tetramethylsilane molecule (experimental value, -1.668 ppm [37]). In brackets, two standard deviations of each dataset across all the structures demonstrate the degree of variance found in the $^{13}$C NMR shifts.

| Species | Average $^{13}$C $\delta_{iso}$ / ppm | |
|---|---|---|
| | 6-311+G(d,p) | aug-pcSseg-2 |
| Bicarbonate | 162(3) | 169(2) |
| Carbonate | 179(5) | 186(5) |

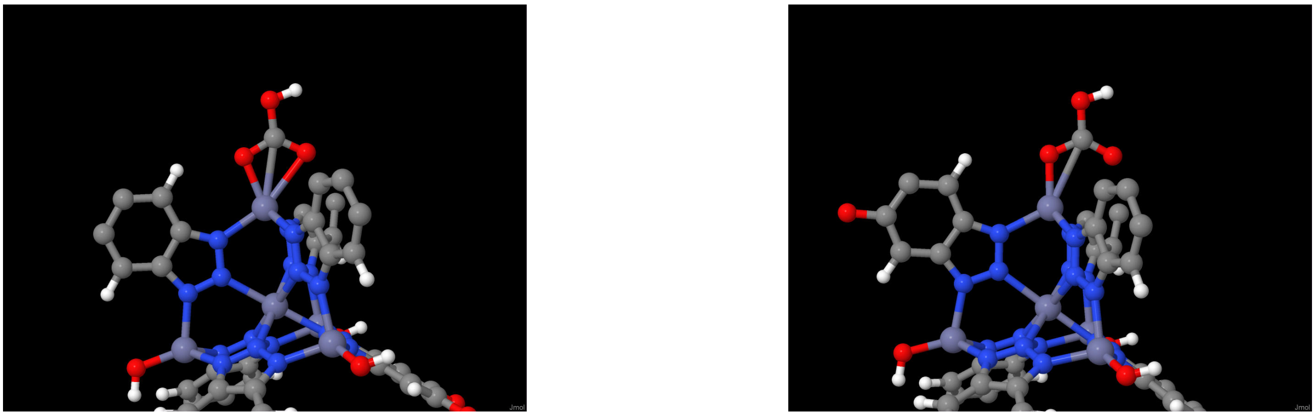

**(a)** Mode A

**(b)** Mode B

**Figure S10.** Geometry optimised configurations of MFU-4l with one hydroxyl site bound with bicarbonate to simulate $CO_2$ adsorption. Mode-A has a more symmetric divalent bond, whereas mode-B has an asymmetric monovalent metal-bicarbonate bond. Mode A has a lower energy by 1.7 kJ mol$^{-1}$ and agrees with the literature minimum energy structure. [1,38]

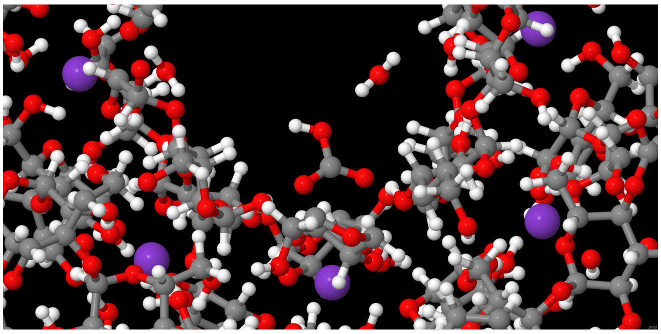

**Figure S11.** Depiction of the model crystal file used for the plane-wave basis set bicarbonate calculation for $KHCO_3$-CD-MOF used in Fig. 1, produced from the .cif file provided in Reference [2]. [2] A single hydrating $H_2O$ molecule can be seen hydrogen-bonded to the hydroxyl group of the carbonate group.

## Further NMR and MLFF molecular dynamic simulations

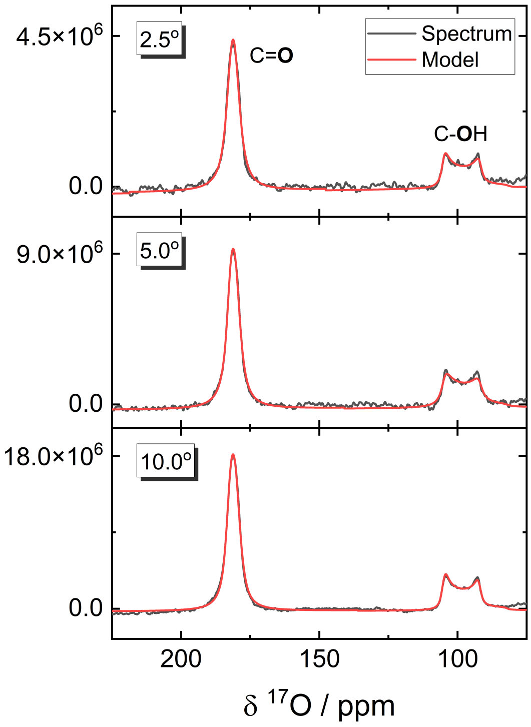


**Figure S12.** Three quantitative $^{17}O$ NMR spectra (23.5 T, 20 kHz MAS, 2.6h, one-pulse) of MFU-4l dosed with $C^{17}O_2$. Pulse angles were 2.5, 5 and 10° relative to the $H_2O$(l) optimised 90° pulse reference, ensuring a quantitative regime was reached.[26] The respective carbonyl : hydroxyl peak ratios of the fitted lineshape integrals are 2.3, 2.1 and 2.3.

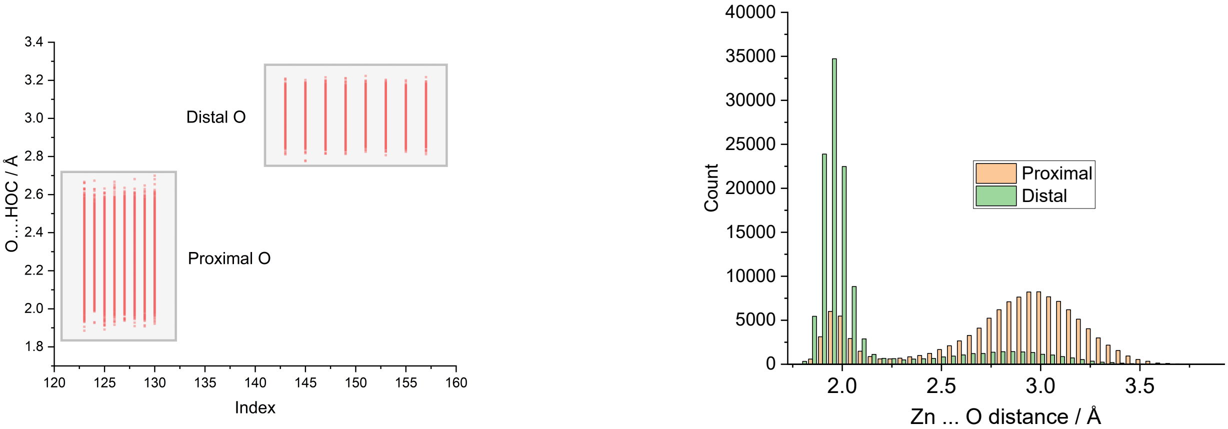


**(a)** **(b)**

**Figure S13.** Processed atomic distance data from the MLFF 2 ns simulation of MFU-4l. **(a)** Shows the carbonyl to hydroxyl hydrogen distances of the bound bicarbonate motif against atom index. There is clear chemical distinction between the proximal and distal environments on this timescale. **(b)** The binned distance data between the respective distal and proximal environments and the Zn centre. The ratio between the distal : proximal oxygen being bound to the Zn is found at 83 : 17.

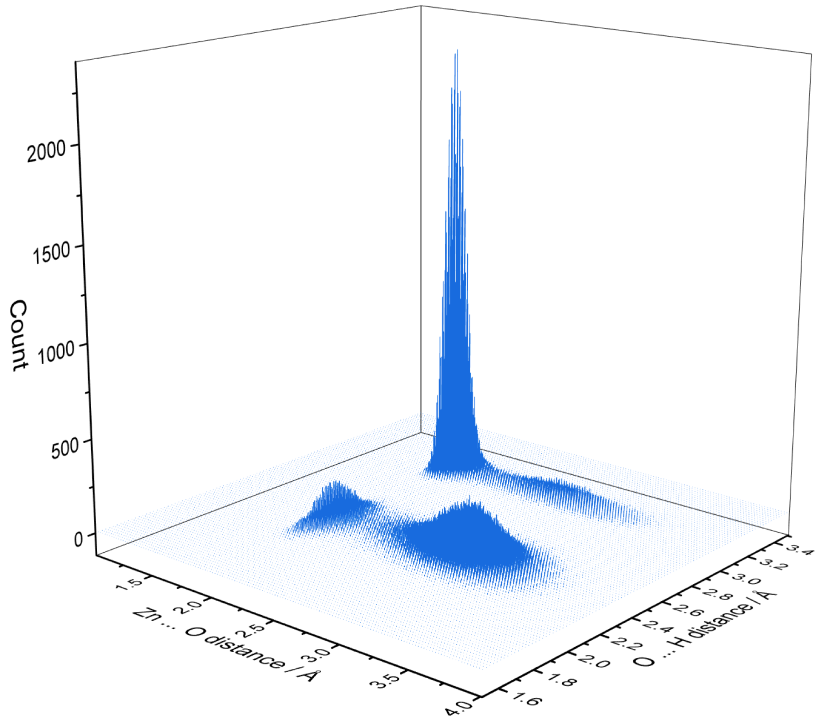


**Figure S14.** The same data as in Fig. S13 plotted as 3D binned count data with respect to zinc-oxygen and carbonyl to hydroxyl proton distances.

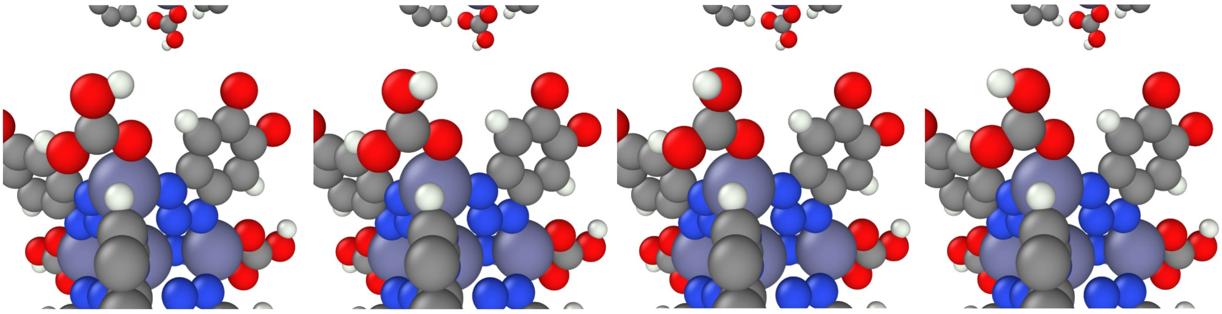

**Figure S15.** Snapshots of the energy barrier pathway for the calculated Fig. S16 'proton-hop' trajectory.

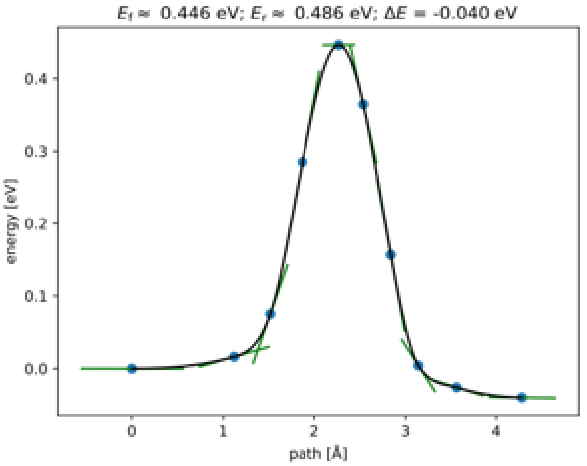


**Figure S16.** The energy barrier pathway calculated via a climbing-image nudged elastic band method for hypothesised 'proton-hop' motion in MFU-4l (see Fig. S15). The slight asymmetry of the profiles is due to subtle differences in the surrounding framework. The larger barrier height of 0.49 eV gives rise to a rate constant of 2.4 x $10^5$ Hz under the harmonic transition state approximation (T = 308 K), which is utilised in the EXPRESS simulations in the main text.

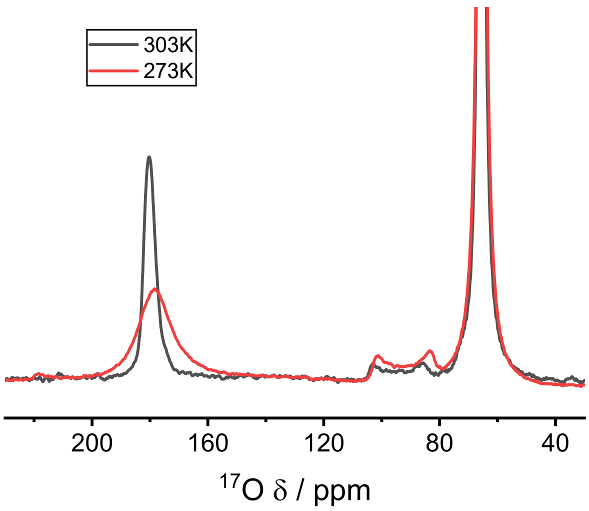


**Figure S17.** Variable temperature (VT) NMR results taken for MFU-4l (20.0 T, 12.5 kHz (303K) and 13.5 kHz (273), 0.1 h (303 K) and 4.7 h (273 K), one-pulse). The significant change in lineshape, with a relatively small temperature swing, demonstrates qualitatively that the chemical exchange is at the edge of the intermediate regime. Temperatures are quoted as non-adjusted probe temperatures. A full quantitative experimental determination of rate constant by VT NMR is beyond the scope of this work.

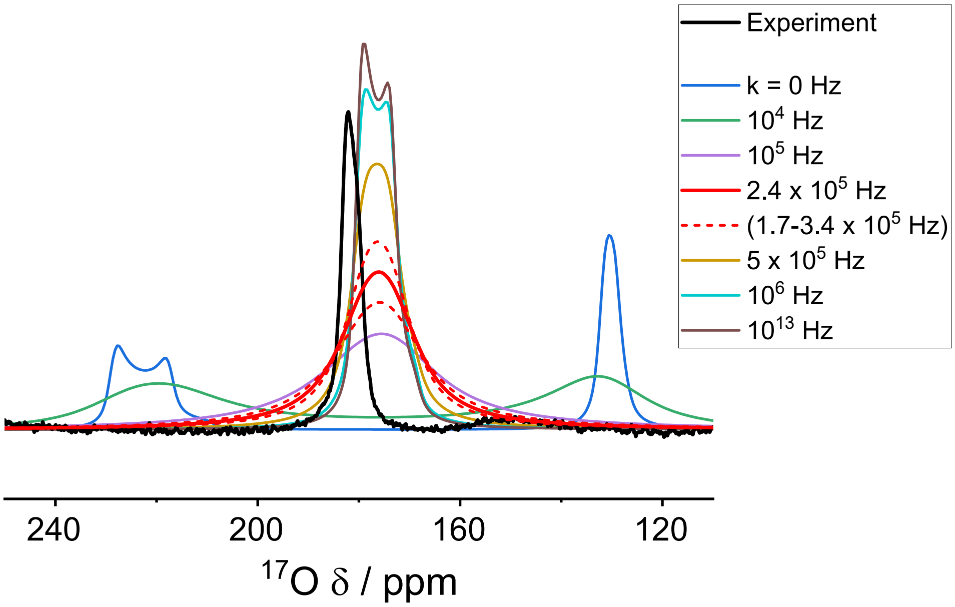


**Figure S18.** A full range of EXPRESS simulated rate constants in comparison to a reproduction of the experimental (black) and modelling (red) data of MFU-4l MOF dosed with $C^{17}O_2$ from Fig. 3**D**. The dashed red lines illustrate a rate constant range calculated from the same barrier (0.49 eV) as the k = 2.4 x $10^5$ Hz but with a ± 5 K temperature difference used as input for the transition state theory.

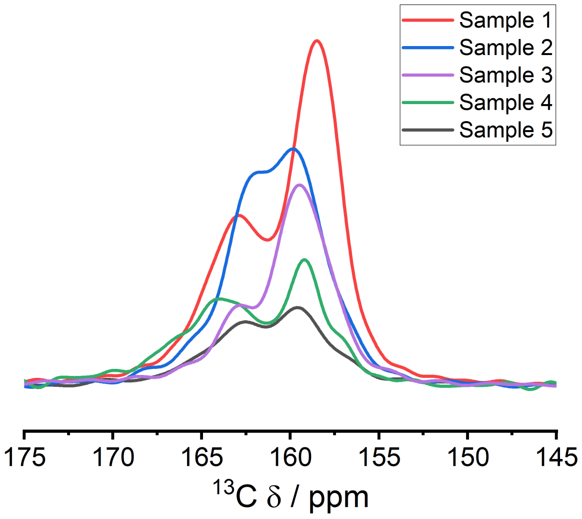


**Figure S19.** $^{13}C\{^1H\}$ CP MAS spectra (9.4 T, 12.5kHz MAS. Hartmann-Hahn matching was achieved with a 90-100% ramping pulse in the proton channel, contact time = 2.5 ms) in the chemisorbed $CO_2$ region of 5 different $KHCO_3$-CD-MOF samples (scaling is arbitrary for clarity). The two chemisorbed environments in ranges 158.5 - 159.8 ppm and 162.0 - 164.0 ppm are observed in all spectra.

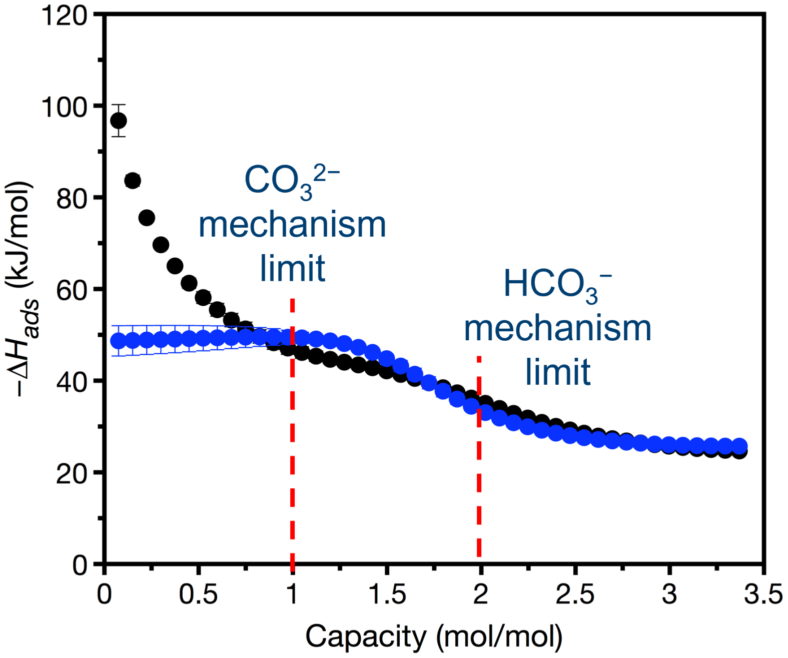


**Figure S20.** Heats of adsorption data of $KHCO_3$-CD-MOF derived from $CO_2$ isotherm data taken at three temperatures (303, 313, 323 K)[2]. Blue is a dual-site Langmuir fitting and black is a dual-site Freundlich-Langmuir fitting. This figure is adapted with the author's permission from *Zick et al.* 2022 (Figure S53) with a corrected scaling to the x-axis (© 2022 Wiley-VCH GmbH). As there are 2 moles of $OH^-$ per mole of MOF,[39] the respective mechanistic limits are indicated. The observed point of inflection at $1.5 \pm 0.05$ mol/mol for the chemisorption to physisorption regime switch over would suggest a theoretical carbonate : bicarbonate ratio of $\approx$ 1 : 1.6 - 2.4.

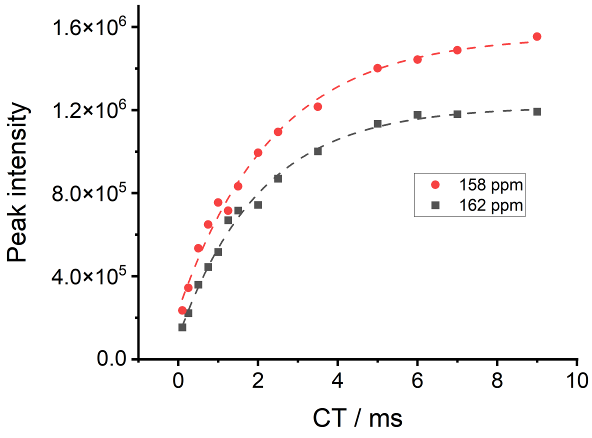


**Figure S21.** CP build up curves for the two chemisorbed $CO_2$ environments of $KHCO_3$-CD-MOF taken at (CT = 0.1-10 ms, 9.4 T and 12.5 kHz MAS, 2.5h, under Hartmann-Hahn matching.[40] A ramped matching pulse from 90-100% was used on the proton channel). $T_{HC} = 2.4 \pm 0.2$ ms and $2.1 \pm 0.1$ ms for the the 159 and 163 ppm peaks, respectively. These cross-polarisation build up rates fit in the expected range of non-directly hydrogen bound carbon environments of $T_{CH} = 0.5 - 3$ ms.[41,42] The similar $T_{CH}$ values indicate a similar type of hydrogen environment and similar carbon species, however, the possibility of a labile, dynamic hydroxyl group of a bicarbonate species and a strongly hydrogen bonded carbonate species cannot be fully distinguished by CP alone (especially given the large number of protons in the CD system).

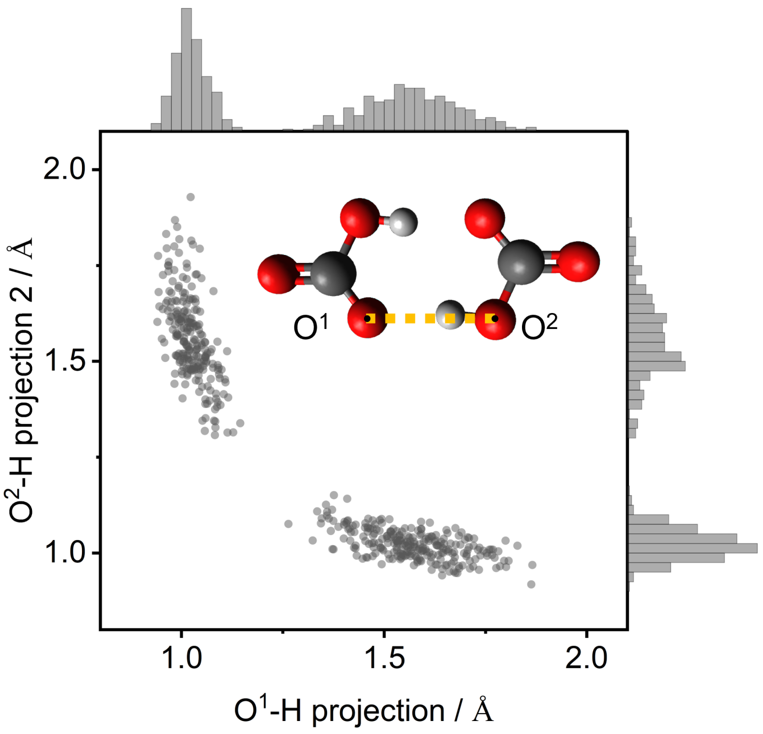


**Figure S22.** The same data as presented in Fig. 5**C** with the correlated projection vectors from both oxygen atoms, $O^1$ and $O^2$, plotted against each other. This demonstrates the symmetry of proton movement over the MD trajectory - justifying the simulation length as sufficient.

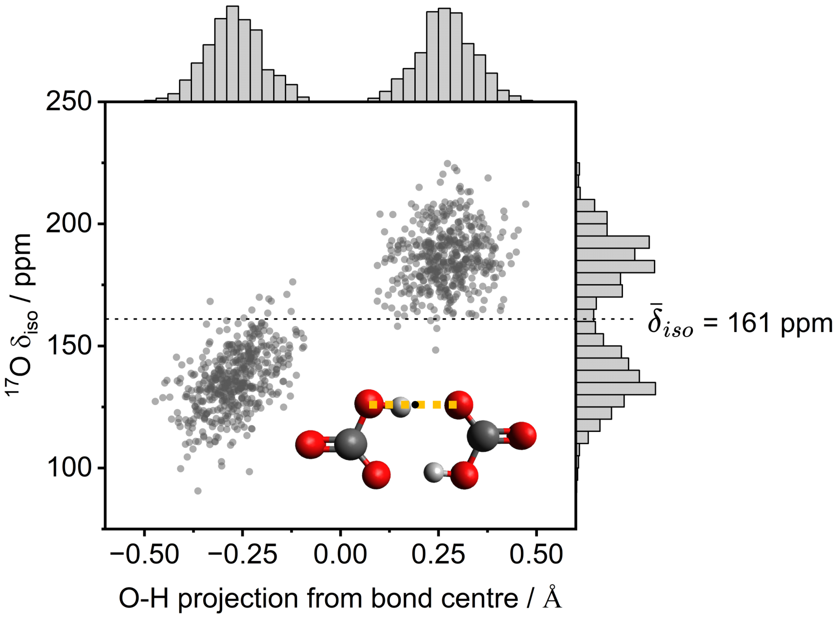


**Figure S23.** Data replicated from Fig. 5**C** with the origin taken at the centre of the oxygen-oxygen vector.

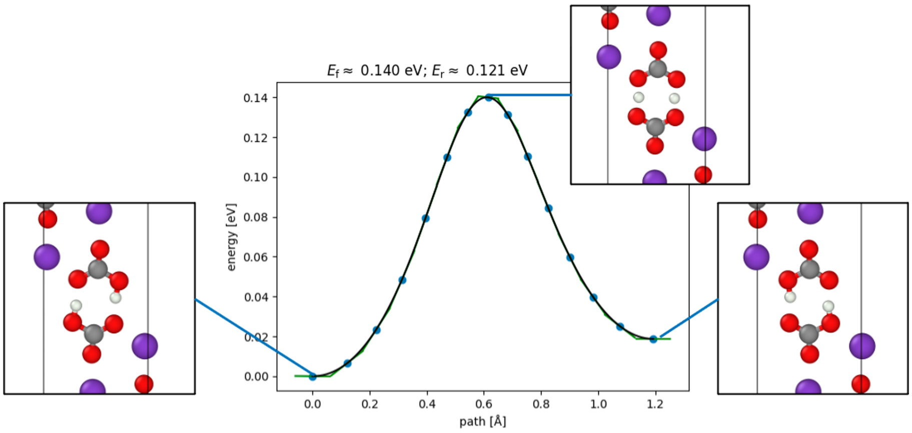


**Figure S24.** A plot of the calculated energy profile of the concerted proton hop within the $KHCO_3$ dimer structure. The barrier height gives a derived rate of proton hop of 140 GHz.

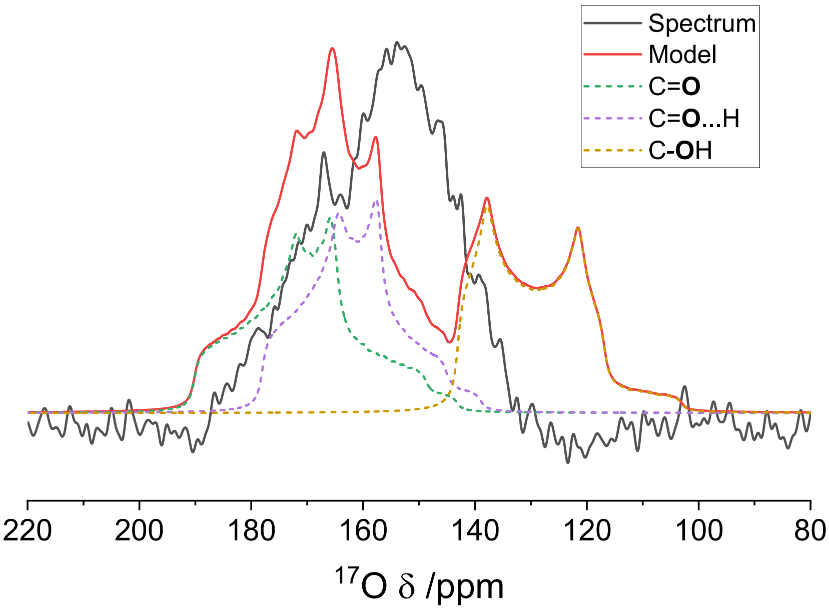


**Figure S25.** A comparison of the 23.5 T $^{17}O$ NMR experimental data for $KHCO_3$(s) from this work with the experimental fit results from *Peach et al.*.[43] There is a clear discrepancy in the low shift region from 110-140 ppm indicating a different dynamic system for the hydroxyl environment potentially due to different phases of $KHCO_3$ under the MAS conditions.[17,44–46]

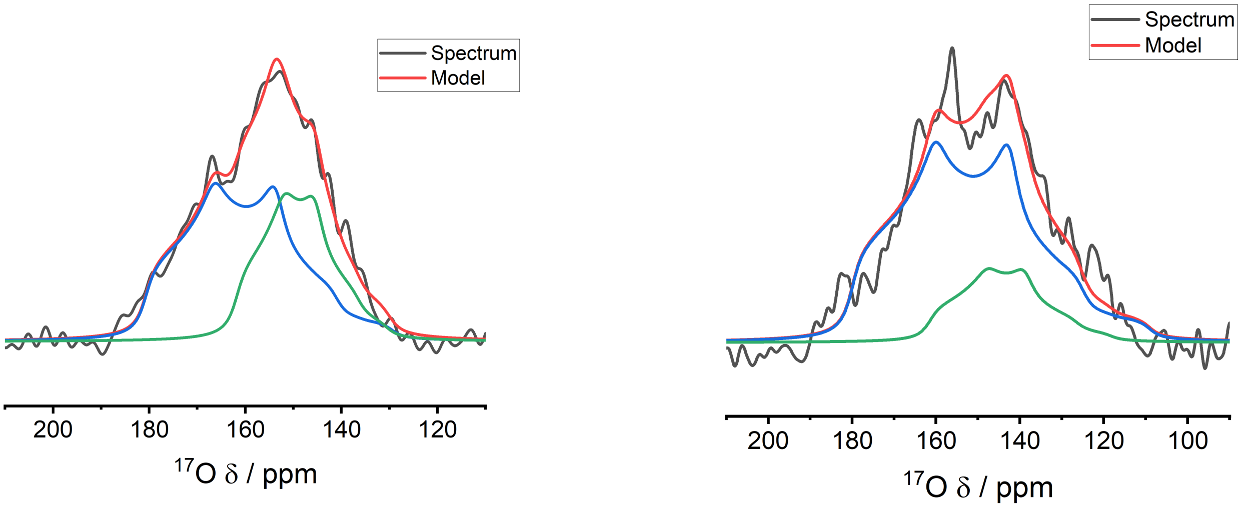


**(a)**

**(b)**

**Figure S26.** $^{17}$O NMR spectra of $KHCO_3$(s) taken at 23.5 T, **(a)**, (20 kHz MAS, 33.0 h, hahnecho) and 20.0 T, **(b)**, (12.5 kHz MAS, 23.3 h, hahnecho). The modelled lineshapes are produced from a multi-field fit of both spectra in ssNake.[29] Fit parameters were obtained over a range of line broadenings and initial parameters (giving a 2$\sigma$ error in brackets): $\delta_{iso}$ = 182.6(3) ppm, $C_Q$ = 7.9(1) MHz, $\eta_Q$ = 0.49(1) and 162.6(5) ppm, 6.1(1) MHz, 0.57(3).

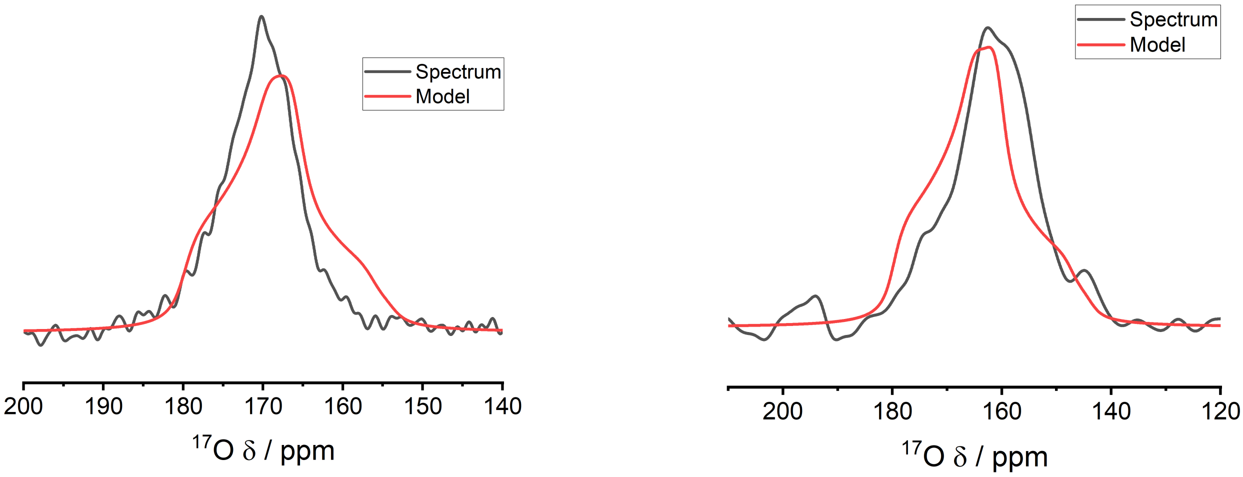


**(a)**

**(b)**

**Figure S27.** $^{17}$O NMR spectra taken of $K_2CO_3 \cdot 1.5\ H_22O$(s) at 23.5 T, **(a)**, (20 kHz MAS, 39.5 h, hahnecho) and 20.0 T, **(b)**, (12.5 kHz MAS, 15.6 h, hahnecho). The modelled lineshapes are produced from a multi-field fit of both spectra in ssNake.[29] Fit parameters were obtained over a range of line broadenings and initial parameters (giving a 2$\sigma$ error in brackets): $\delta_{iso}$ = 180(1) ppm, $C_Q$ = 5.4(2) MHz, $\eta_Q$ = 0.78(4)

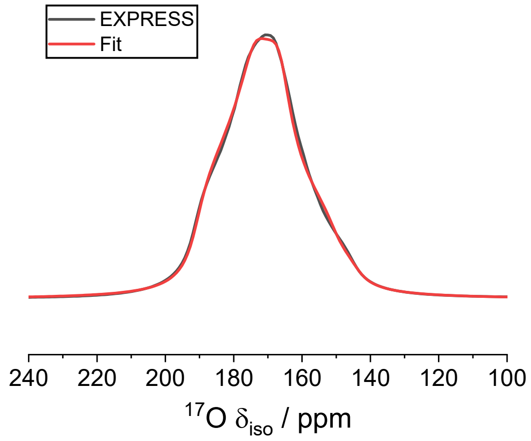


**(a)**

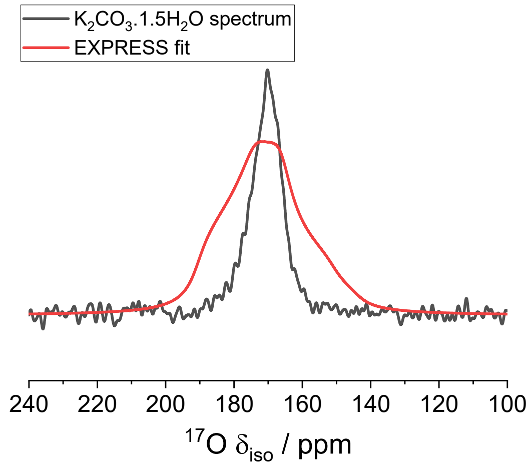


**(b)**

**Figure S28.** Plots of the EXPRESS simulated lineshape of the oxygen environments in $K_2CO_3.1.5H_2O$(s) under exchange. **(a)** a fit (red) of the simulated EXPRESS lineshape (black). Parameters used were generated from the DFT calculation in Table 2 for the three distinct carbonyl environments (1 @ 206 ppm, 2 @ 193 ppm, 3 @ 184 ppm). Euler angles: EFG(1) to crystal field axis (CFA) = [0 0 0], EFG(2) = [-1.47, 59.28, -177.28], EFG(3) = [5.98, 31.85, 175.51], CSA(1) to EFG(1) = [174.91, 89.98, -90.55], CSA(2) to EFG(2) = [176.61, 88.35, -82.78], CSA(3) to EFG(3) = [78.55, 89.15, 178.86]. Rate constant = 1 x $10^4$ Hz. Comparison to the experimental spectra **(b)** shows reasonable match, however the linewidth/$C_Q$ is clearly overestimated.

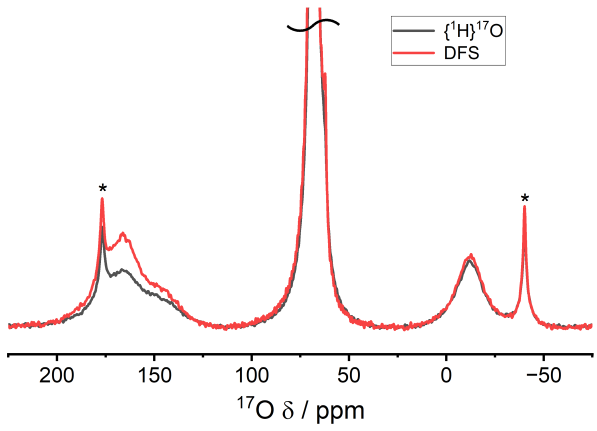


**Figure S29.** An overlay of a {$^1$H} double-frequency sweep (DFS) experiment (DFS is an enhancement technique used to increase signal for environments with a significant quadrupolar interaction)[47,48] with a {$^1$H}$^{17}$O one-pulse experiment of the same number of scans and equivalent pulse parameters (20.0 T, 12.5 kHz MAS, 0.9 h). A frequency sweep of 850-150 kHz at 20 W for 3.2 $\mu$s was used. No enhancement was seen in the mobile $H_2O$ peak at negative ppm and the right-hand side of the chemisorbed $CO_2$ peak at $\approx$ 150 ppm shows limited enhancement relative to the rest of the peak. This is suggested to be a result of the more dynamic nature of the hydroxyl environment assigned at this lower ppm, however further work is required to establish the complete relationship between DFS enhancement and dynamics. Spinning sidebands are denoted by *.

**Table S3.** Comparison of the experimental fit parameters in the carbonyl region of $KHCO_3$-CD-MOF (Fig. 6**A**) and the experimental results for other carbonyl environments in a selection of bicarbonate and carbonate solid and encapsulated crystalline species. * denotes results from this work (see Figs. S26-S27).

| | Expt. CD-MOF fit* **1** - Blue | **2** - Purple | $KHCO_3$* | $K_2CO_3$·1.5 $H_2O$* | $CaCO_3$ [49] | $Li_2CO_3$ [50] | $Li_2CO_3$ [51] | $CO_3^{2-}$ hosted in a cryptand [52] |
|---|---|---|---|---|---|---|---|---|
| $\delta_{iso}$ / ppm | 192 | 181 | 182.6(3) | 180(1) | 204 | 154, 174 | 154.8(5), 171.9(5) | 170 |
| $C_Q$ / MHz | 6.5 | 6.0 | 7.9(1) | 5.4(2) | 6.97 | 7.40(5), 7.20(5) | 7.22(5), 6.90(5) | 7.5 |
| $\eta_Q$ | 0.8 | 0.7 | 0.49(1) | 0.78(4) | ≈ 1 | 0.88(5), 0.95(5) | 0.94(5), 1.00(5) | 0.7 |

## MQMAS and multi-field $^{17}O$ NMR fits

This section describes the fitting procedure utilised to produce the MQMAS and multi-field fit presented in Fig. 6**A** of the $KHCO_3$-CD-MOF dosed with $C^{17}O_2$. MQMAS data is presented in Figs. S30-S31. High resolution was found to be challenging to obtain in the indirect dimension, especially at lower ppm (< 160 ppm). However, weak signal corresponding to the 'dynamic hydroxyl' environment are identifiable when compared to the high-resolution 1D projection (Fig. S31).

The two clear isotropic regions identifiable in the F1 dimension were fit in dmFit [28] with a variety of line-broadening parameters and widths of cross-section through the isotopic dimension to give an uncertainty to the fit (Fig. S32).

The two fits obtained from the MQMAS are compared to the high-resolution 1D data (Fig. S33a). There is a clear absence in the lineshape of fitting for the lower ppm of the peak as a result of the poor signal-to-noise achieved in the MQMAS. However, taking these two environments as a starting point, a third environment, E3, is introduced to account for the missing signal. Similar fitting iterating over various starting conditions and line broadening parameters led to a convergence in fitting given in Fig. S33b.

The final stage of fitting involved confirming the established fit with multi-field NMR data at 20.0 T. The fit parameters from Fig. S33b were then varied, from an initial fixed position, sequentially and cumulatively in integral, $\delta_{iso}$, $C_Q$ and $\eta_Q$. This gave the two final fits as used in Fig. 6**A** and Fig. S34b.

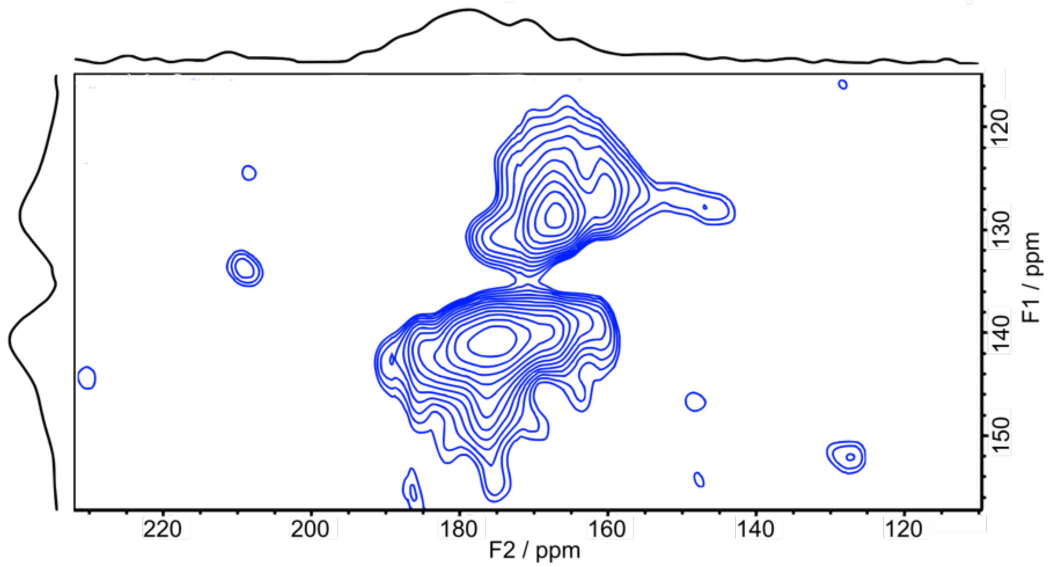


**Figure S30.** An MQMAS spectrum of $KHCO_3$-CD-MOF in the chemisorbed $CO_2$ region (23.5 T, 20kHz, 44.3 h, 3Q-conversion pulses $\omega_1$ = 66.7 kHz, z-filter $\omega_2$ = 20.8 kHz).

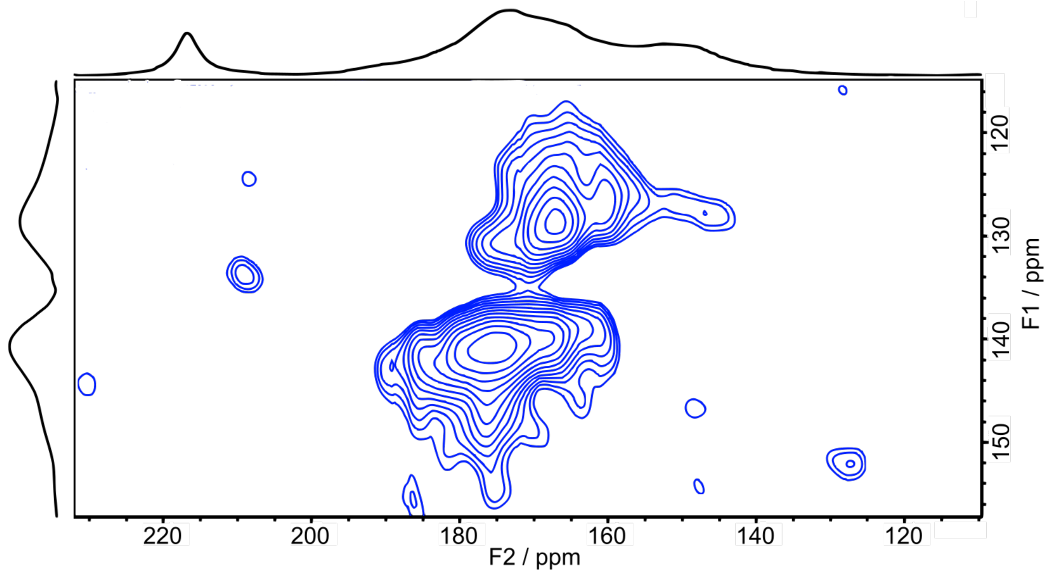


**Figure S31.** The same MQMAS spectrum (23.5 T, 20kHz) as Fig. S30 with the F2 axis projection displayed as the high resolution $\{^{1}\mathrm{H}\}^{17}\mathrm{O}$ NMR spectrum as used in Fig. 6**A**.

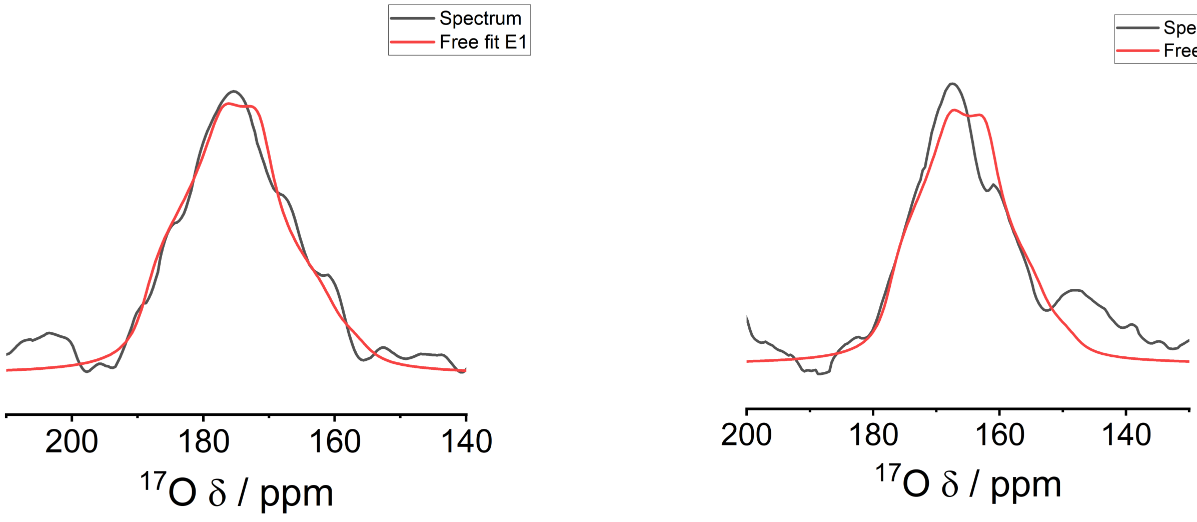


**(a)** **(b)**

**Figure S32.** MQMAS cross-sections fits in the F2 dimension for the two regions of isotropic shift identified in Fig. S30. **(a)** The high-shift environment 1 (E1) has fit parameters ($\delta_{iso}$ = 190.5(8) ppm, $C_Q$ = 6.5(2) MHz, $\eta_Q$ = 0.63(4)) and **(b)** the lower shift E2 has parameters (180.3(8), 6.6(4), 0.54(2)). Fitting uncertainties of $2\sigma$ are given in brackets.

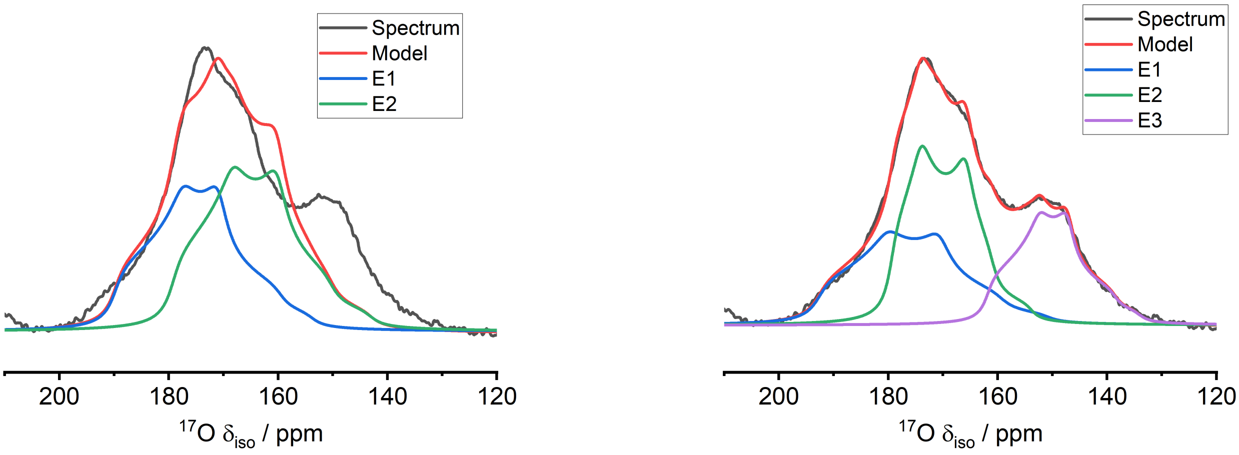


**(a)** **(b)**

**Figure S33. (a)** MQMAS cross-section fits from Fig. S32 fitted in magnitude to high-resolution 1D MAS (23.5 T, 20kHz, 0.9 h, one-pulse) data. After the addition of E3, **(b)**, a high quality fit of the 1D spectrum is produced with parameters: $\delta_{iso}$ = 192(2), 180.6(6),162(1), $C_Q$ = 6.6(6),5.6(1),5.6(2), $\eta_Q$=0.6(1),0.39(4),0.63(1) (line broadening uncertainties of $2\sigma$ are given in brackets).

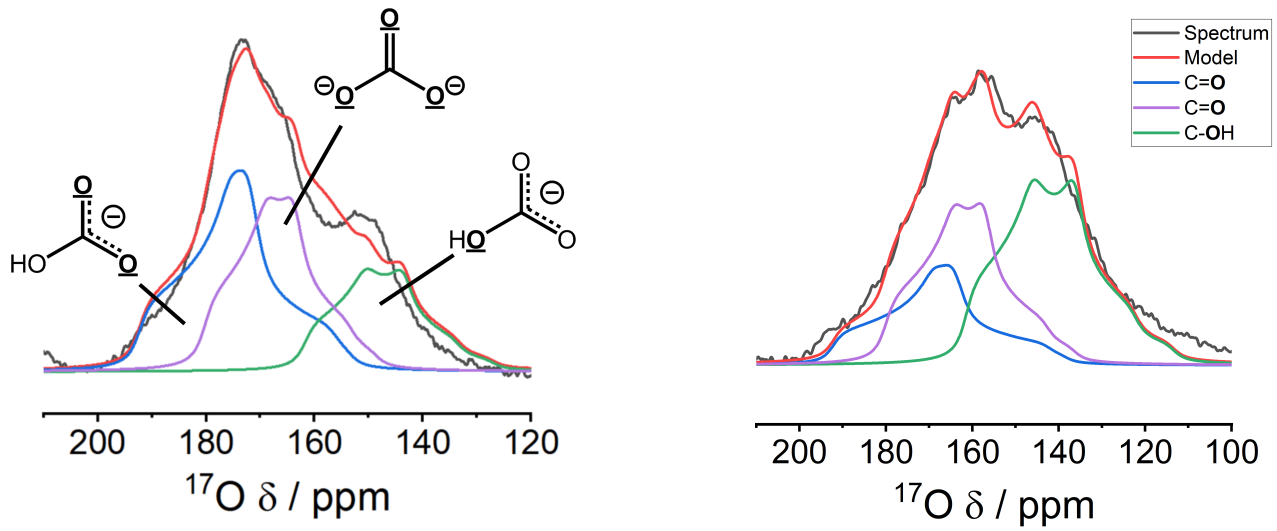


**(a)** **(b)**

**Figure S34.** {$^1$H}$^{17}$O NMR spectra of $KHCO_3$-CD-MOF taken at **(a)** 23.5 T (20 kHz MAS, 0.9 h, one-pulse) and **(b)** 20.0 T (12.5 kHz MAS, 4.8 h, one-pulse), with a multi-field fit as performed in ssNake.[29] The peak ratios, from left to right, are 1.2 : 1 : 0.7 and 0.6 : 1 : 1.3, for 23.5 T and 20.0 T, respectively (see Figs. S38-S39 for quantitative regime establishment). Although the ratios change across the field strengths, the overall ratio between the assigned carbonate oxygens (purple) and bicarbonate oxygens (blue + green) remains constant across the two field strengths (≈ 1 : 1.8-1.9). This is assignable to a smaller absolute frequency difference between the bicarbonate carbonyl (blue) and the 'dynamic hydroxyl' (green) at 20.0 T that are hypothesised to be involved in slow dynamic exchange, thus affecting peak intensities.

## Additional NMR, quantification and CD-MOF MLFF structures

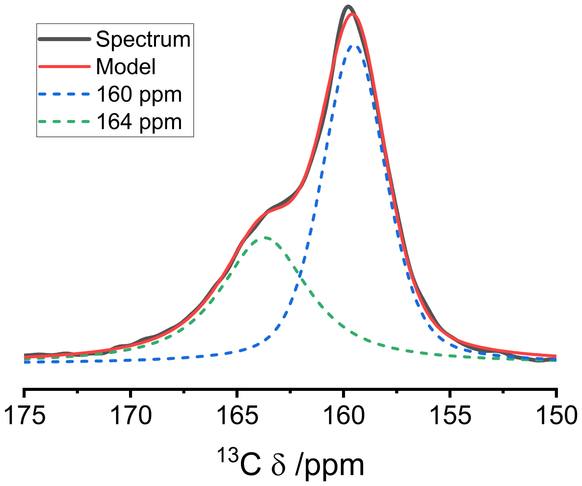


**Figure S35.** A {$^1$H}$^{13}$C NMR spectrum of $KHCO_3$-CD-MOF (9.5 T, 12.5 kHz, 16 h) taken under quantitative experimental conditions ($T_1$ = 60 s, recycle delay = 300 s). The absolute integrals of the fitted peaks at 160 and 164 ppm, respectively, are 434480908.66 and 267300226.05 giving an approximate 1 : 1.6 ratio (fitted in dmFit with a free parameter Lorentzian/Gaussian fit. G/L ratio parameters were 0.6 and 0.15, respectively).

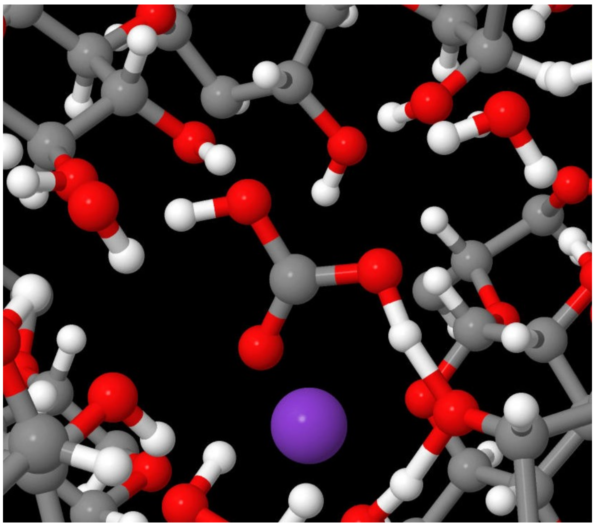

(a)

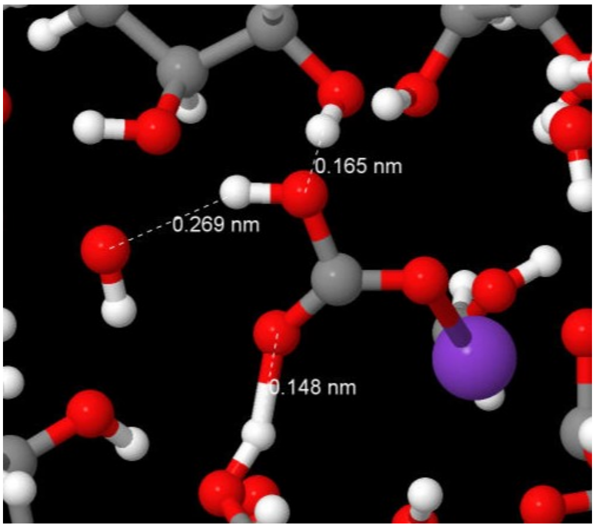


(b)

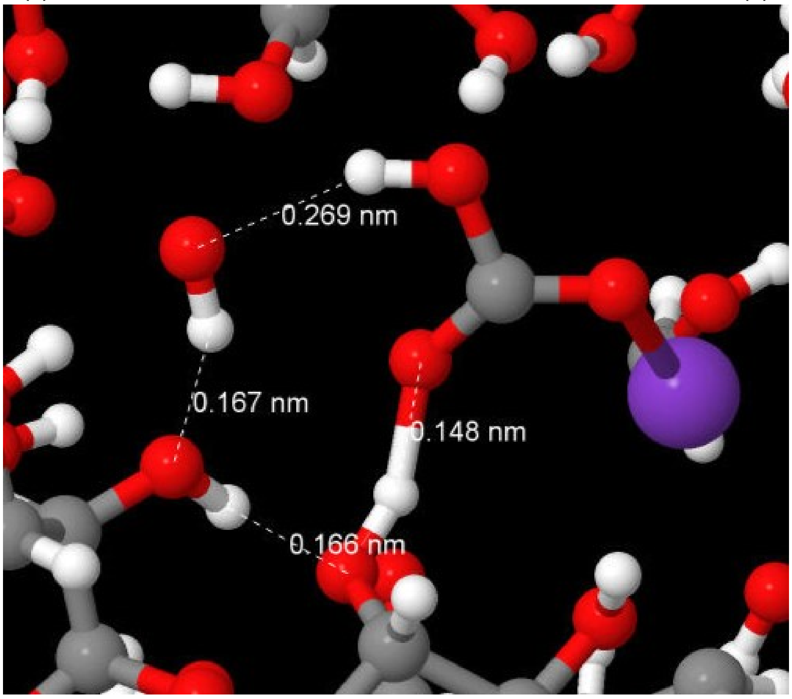


(c)

**Figure S36.** Three snapshot frames taken from a MACE-MP-0 foundation model force-field [23] MD simulation of $KHCO_3$-CD-MOF. **(a)** shows a carbonic acid type moiety where carbonyl-to-hydroxyl type dynamics have been seen. In **(b-c)** (the same frame) two possible mechanisms for hydroxyl-to-carbonyl and carbonyl-to-hydroxyl dynamics are envisaged through proton movements along the depicted bonds lengths. Both mechanisms involve a facilitating adjacent hydroxide anion that has migrated from a nearby unsaturated potassium site. All the proposed mechanisms maintain one carbonyl not involved with dynamics. **(c)** replicates Fig. 6**B** with the proposed proton motion corroborated from a calculated perturb structure energy minimisation pathway.

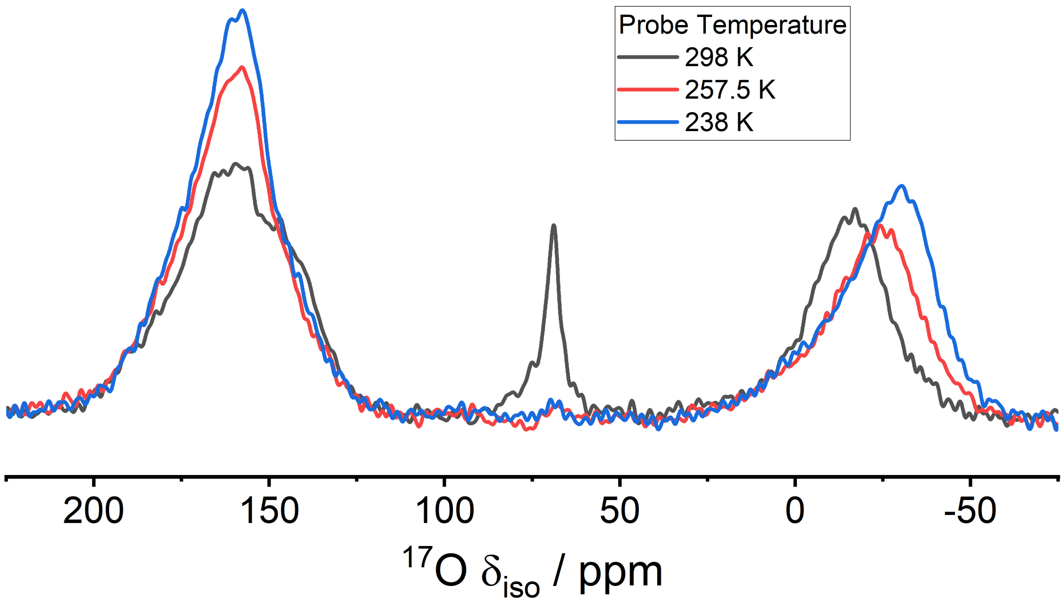


**Figure S37.** Variable temperature NMR measurements of $C^{17}O_2$-dosed $KHCO_3$-CD-MOF (12.5 kHz, 20.0 T, 0.8 h, one-pulse). Disappearance of the physisorbed $CO_2$ peak and corresponding enhancement of the chemisorbed peak is seen, as expected as temperature is lowered. However, this change in equilibrium is challenging to deconvolute from any variation in dynamics and any other contributions that lead to the apparent narrowing of the spectral peak. Additionally, the $H_2O$ peak is observed to shift to more negative ppm with lower temperature, again with the possibilities of physical effects (state change and dynamics) and chemical equilibrium ($H_2O/OH^-$) likely contributing. Further work is required to assign these spectral changes fully.

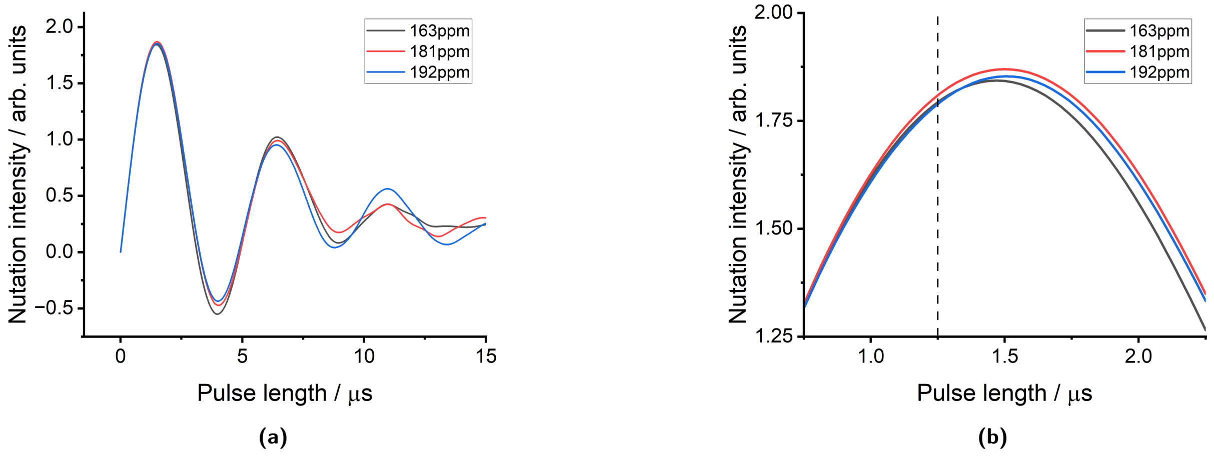


**Figure S38.** $^{17}$O NMR nutation curves simulated in SIMPSON[53] for the experimental parameters (see Tables 1-2) derived from fits of the 23.5 T spectrum of the $KHCO_3$-CD-MOF ((**b**) is a zoomed subsection of (**a**)). An RF-field of 66.666 kHz was used and the 3 curves represent the 3 environments as defined by the experimental fit parameters given in Fig. 6**A** and S34b. A 1% deviation in nutation intensity is found at the pulse length of 1.25 $\mu$s (dashed line) that was used experimentally.

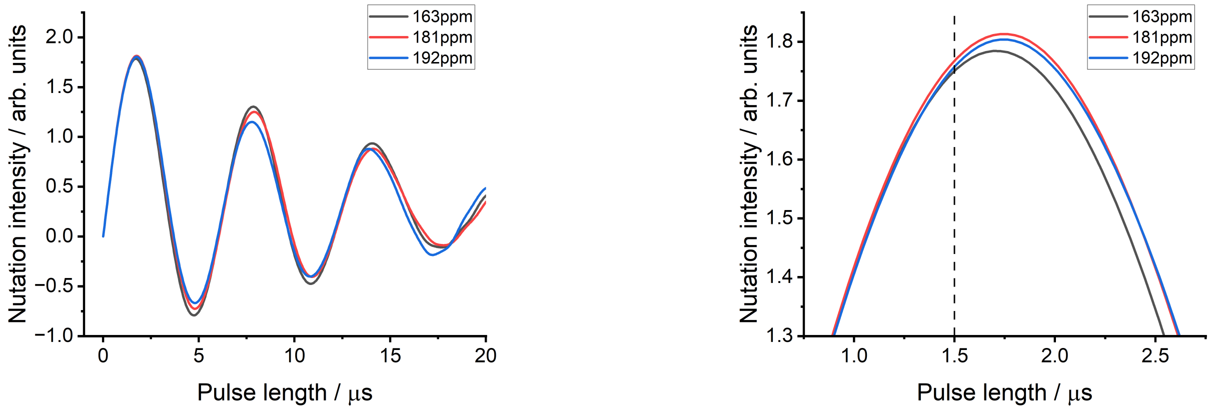


**(a)** **(b)**

**Figure S39.** $^{17}$O NMR nutation curves simulated in SIMPSON[53] for the experimental parameters (see Tables 1-2) derived from fits of the 20.0 T spectrum of the $KHCO_3$-CD-MOF (**(b)** is a zoomed subsection of **(a)**). An RF-field of 55.556 kHz was used and the 3 curves represent the 3 environments as defined by the experimental fit parameters given in Fig 6**A** and S34b A 1% deviation in nutation intensity is found at the pulse length of 1.5 $\mu$s (dashed line) that was used experimentally.

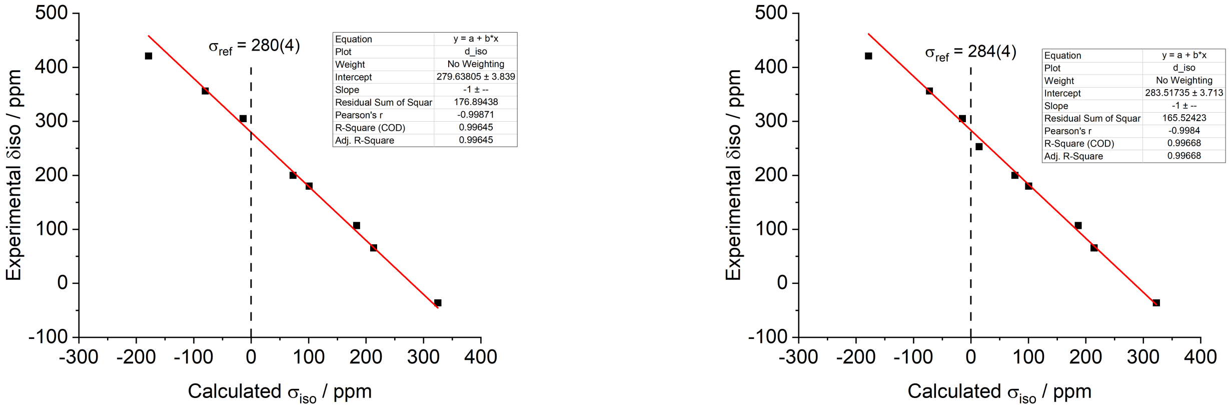


**(a)** **(b)**

**Figure S40.** Plots to determine $\sigma_{ref}$ values for cluster calculations. Calculated chemical shielding values in aug-pcsSeg-2 (**a**) and 6-311+G(d,p) (**b**) basis sets are plotted against experimental values (see section *Static DFT methods*) of the same species to give a y-intercept value of $\sigma_{ref}$ for the fixed-gradient ($-1$) fit line. It is noted that a free fit of the data, without a fixed gradient, also returned a gradient of $-1$ within error ((a) -0.9669 ± 0.03493 and (b) -0.98896 ± 0.0395).

## Materials characterisation

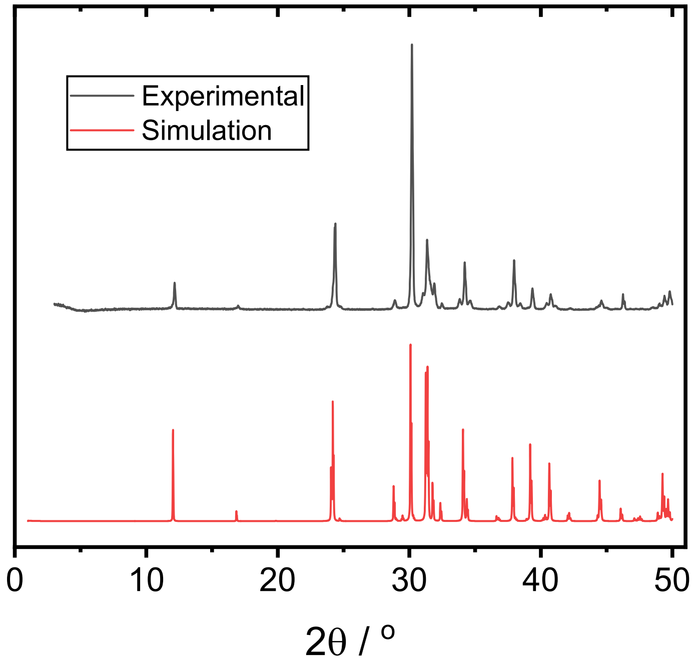


**Figure S41.** PXRD pattern (black) taken of the same chemical sample of $KHCO_3$(s) as was used in obtaining the $^{17}$O NMR spectra in Fig. S26. Comparison to the simulated powder pattern (red) generated in VESTA [54] from the crystal structure of Allan *et al.*. [17]

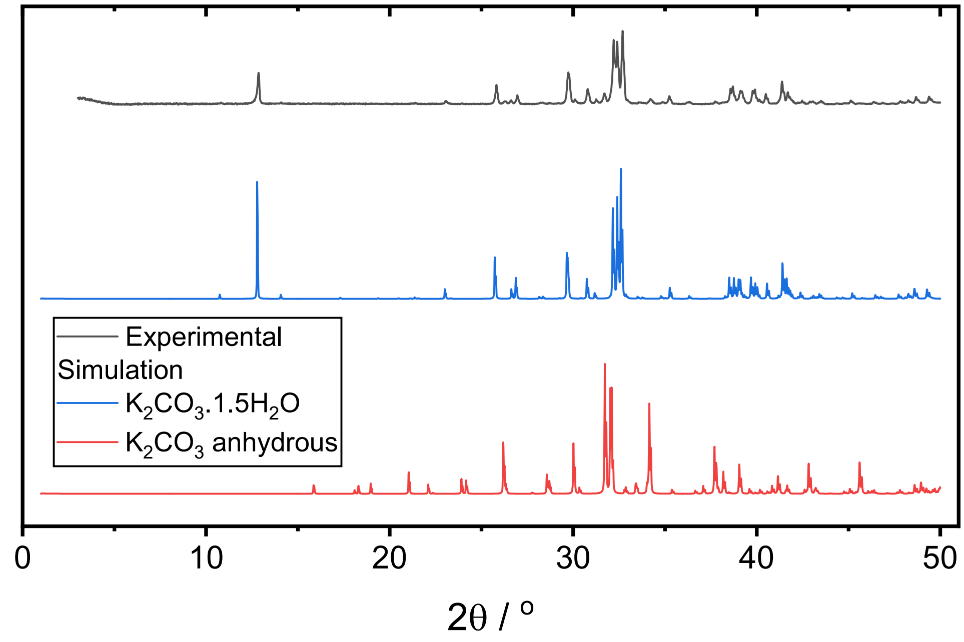


**Figure S42.** PXRD pattern (black) taken of the same chemical sample of $K_2CO_3$(s) as was used in obtaining the $^{17}O$ NMR spectra in Fig. S27. Comparison to simulations of the crystal structures of anhydrous $K_2CO_3$ (red), Gatehouse and Lloyd,[55] and $K_2CO_3{\cdot}1.5\ H_2O$ (blue), Skakle *et al.*,[56] reveals the experimental sample was in the hydrated form.

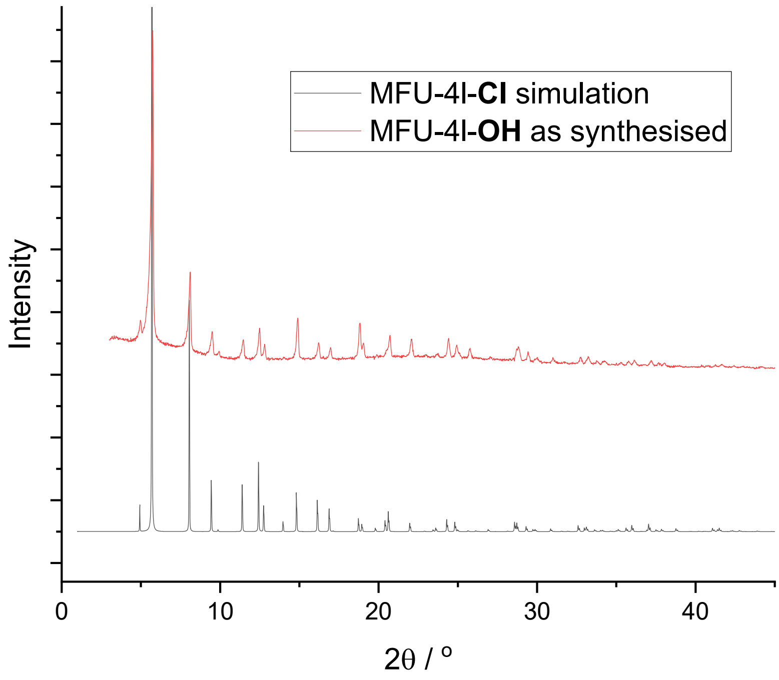


.

**Figure S43.** PXRD pattern of the MFU-4l sample (red) used in Figs. 2-3 showing good crystallinity and agreement with literature simulation (black) of the pre-exchanged chloride MOF of the same structure.[1,16] The simulation is generated in VESTA.[54]

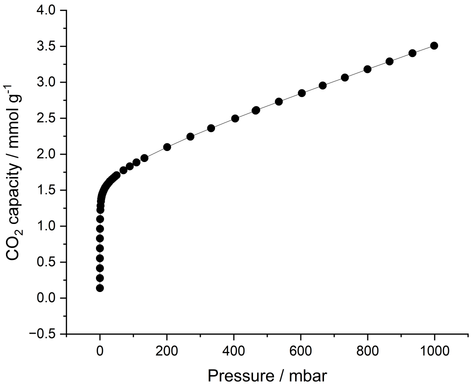


**(a)**

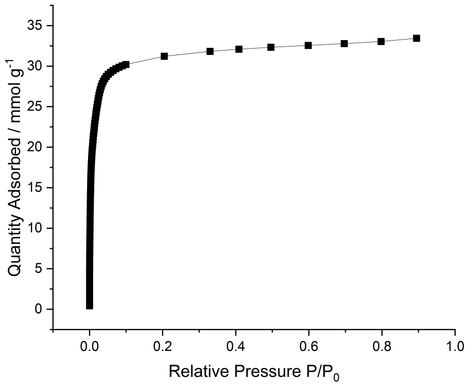


**(b)**

**Figure S44.** Carbon dioxide **(a)** and nitrogen **(b)** adsorption isotherm data for the MFU-4l sample. The $CO_2$ isotherm (taken at 300 K) shows characteristic steep uptakes at low pressure and a capacity of 1 bar of 3.5 mmol $g^{-1}$ in agreement with literature 2.8-3.4 mmol $g^{-1}$.[1,57] The BET surface area of this sample was 2727 $m^2$ $g^{-1}$, taken at 77 K, also in line with the literature reported values of 2613-2739 $m^2$ $g^{-1}$.[1,57]

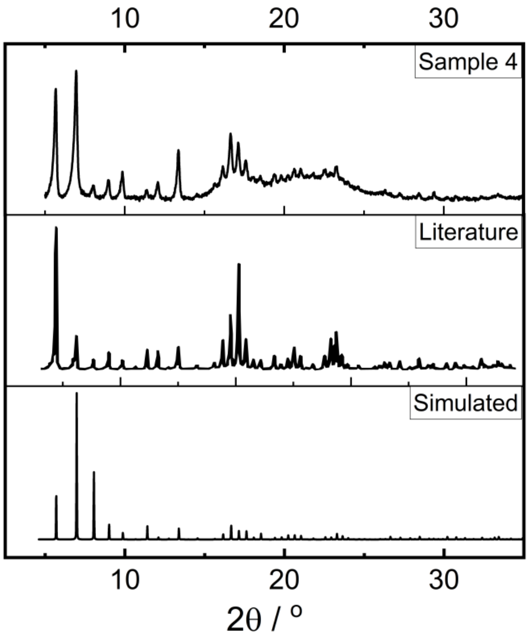


**(a)**

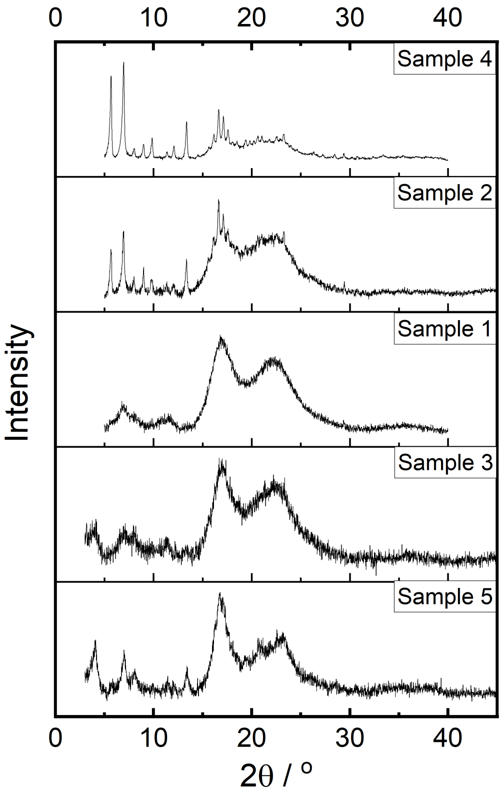


**(b)**

**Figure S45.** PXRD patterns of the $KHCO_3$-CD-MOF samples. Comparison to a VESTA[54] simulation and literature[2] pattern is shown in Fig. **(a)**. Comparison of the same five $KHCO_3$-CD-MOF samples as shown in Fig. S19 under $^{13}C$ NMR is shown in **(b)**. Despite clear variations in sample quality and amorphous character contributions, powder patterns share characteristic features.

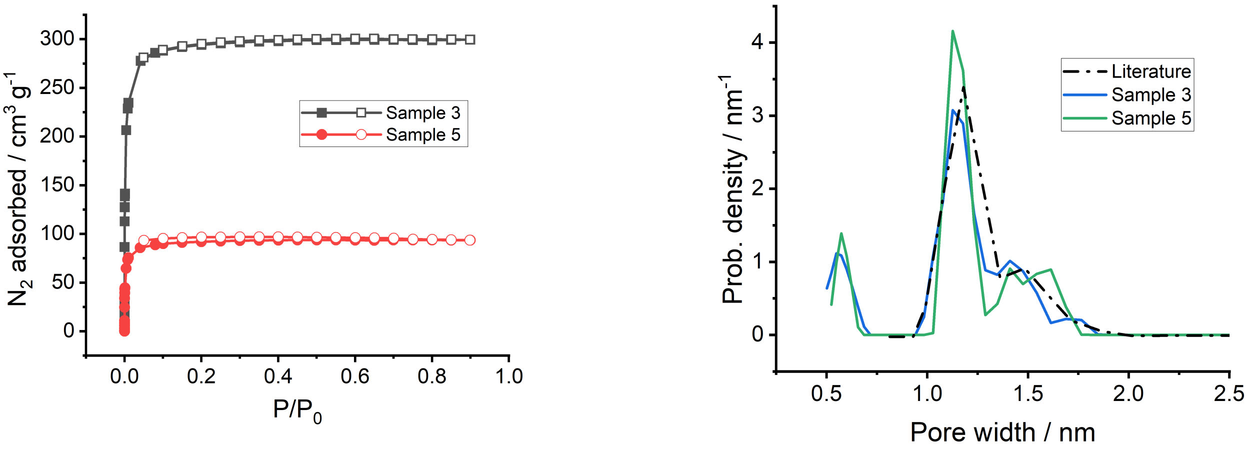


**(a)**

**(b)**

**Figure S46.** Results of $N_2$ isotherm data for two of the freshly synthesised $KHCO_3$-CD-MOF samples. Filled symbols represent adsorption, blank symbols represent desorption. The raw adsorption isotherms, **(a)**, gave BET areas of 1184(1.4) and 365.7(5) $m^2g^{-1}$ for samples 3 and 5, respectively. This compares to the literature value of 1220 $m^2g^{-1}$,[39] which is a good match for sample 3 despite its apparent amorphous character from the PXRD, Fig. S45b. Pore size distribution plots calculated by the NLDFT method (slit-pore) of the same two isotherms are plotted in **(b)** and compared to a literature distribution taken from Patel *et al.* who used an unspecific NLDFT model.[58] The apparent consistency of pore size distribution, despite BET area differences, helps gives validity to mechanistic study of these materials.

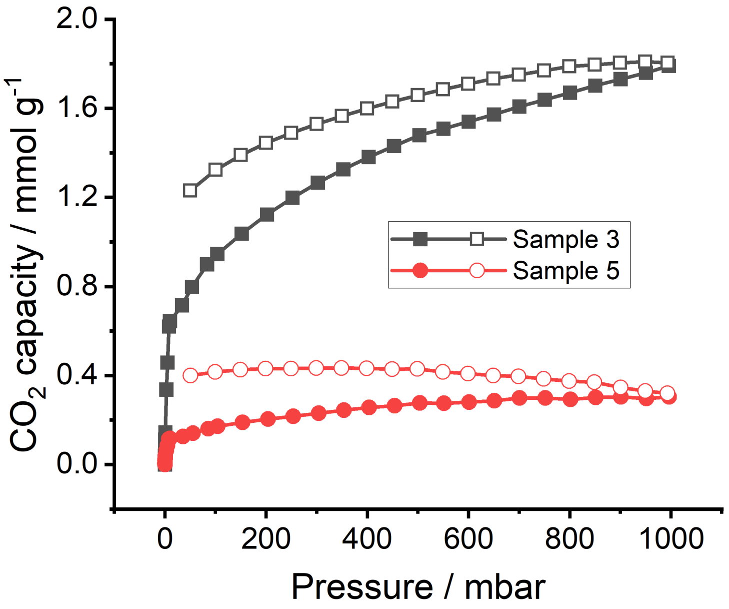


**Figure S47.** $CO_2$ sorption isotherms of fresh samples 3 and 5. Filled symbols represent adsorption, blank symbols represent desorption. The maximum capacities at 1 bar of 1.8 mmol $g^{-1}$, sample 3, and 0.3-0.4 mmol $g^{-1}$, sample 5, demonstrate that despite unexpected amorphous character significant $CO_2$ uptake is still possible (in the case of sample 3, up to 75% of the literature value of 2.42 mmol $g^{-1}$)[2]).

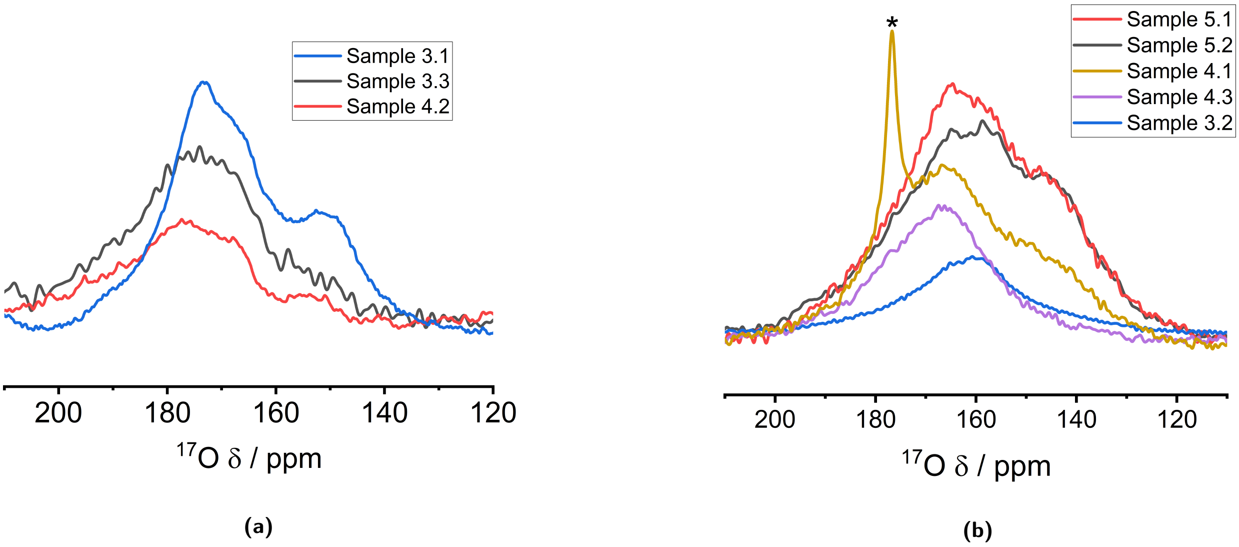


**Figure S48.** Repeated $^{17}$O NMR measurements of $C^{17}O_2$-dosed $KHCO_3$-CD-MOF for a range of samples at 23.5 T, **(a)**, (20 kHz MAS, one-pulse{$^1$H}) and 20.0 T, **(b)**, (20 kHz MAS, one-pulse{$^1$H}). The '.1'-'.3' labels represent the same sample measure at a chronologically different time: for 3.1 to 3.2 (+1 week) to 3.3 (≈ +3 months); 4.1 to 4.2 (≈ +1 month) to 4.3 (≈ +2 months); and 5.1 to 5.2 (≈ +1 month). It is clear on the 3-month timescale that degradation of sample 4 has occurred leading to loss of resolution in the spectrum. The same is hypothesised to have occurred in sample 3.2 at 20.0 T, thus the multi-field fit in Fig. S34 utilises data from the higher resolution sample 5.

**Table S4.** Explicit tabulation of which $KHCO_3$-CD-MOF samples were used in each of the NMR spectra present in this work.

| Figure | Sample(s) |
|---|---|
| Figs. 4**A**, S35 | 1 |
| Fig. S21 | 2 |
| Figs. 4**C-D**, 6, S30-S34 | 3.1 |
| Fig. S29 | 4.1 |
| Fig. 4**B** | 4.2 |
| Fig. S34, S37 | 5.2 |

# References


[1] Z. Cai, C. E. Bien, Q. Liu, C. R. Wade, *Chem. Mater.* **2020**, *32*, 4257.
[2] M. E. Zick, S. M. Pugh, J.-H. Lee, A. C. Forse, P. J. Milner, *Angew. Chem. Int. Ed.* **2022**, *61*, e202206718.
[3] M. J. Frisch, G. W. Trucks, H. B. Schlegel, G. E. Scuseria, M. A. Robb, J. R. Cheeseman, G. Scalmani, V. Barone, G. A. Petersson, H. Nakatsuji, X. Li, M. Caricato, A. V. Marenich, J. Bloino, B. G. Janesko, R. Gomperts, B. Mennucci, H. P. Hratchian, J. V. Ortiz, A. F. Izmaylov, J. L. Sonnenberg, Williams, F. Ding, F. Lipparini, F. Egidi, J. Goings, B. Peng, A. Petrone, T. Henderson, D. Ranasinghe, V. G. Zakrzewski, J. Gao, N. Rega, G. Zheng, W. Liang, M. Hada, M. Ehara, K. Toyota, R. Fukuda, J. Hasegawa, M. Ishida, T. Nakajima, Y. Honda, O. Kitao, H. Nakai, T. Vreven, K. Throssell, J. A. Montgomery Jr., J. E. Peralta, F. Ogliaro, M. J. Bearpark, J. J. Heyd, E. N. Brothers, K. N. Kudin, V. N. Staroverov, T. A. Keith, R. Kobayashi, J. Normand, K. Raghavachari, A. P. Rendell, J. C. Burant, S. S. Iyengar, J. Tomasi, M. Cossi, J. M. Millam, M. Klene, C. Adamo, R. Cammi, J. W. Ochterski, R. L. Martin, K. Morokuma, O. Farkas, J. B. Foresman, D. J. Fox, Gaussian 16 Rev. C.01 **2016**, gaussian Inc. Wallingford CT.
[4] F. Jensen, *J. Chem. Theory Comput.* **2015**, *11*, 132.
[5] M. D. Hanwell, D. E. Curtis, D. C. Lonie, T. Vandermeersch, E. Zurek, G. R. Hutchison, *J. Cheminf.* **2012**, *4*, 17.
[6] W. W. Rudolph, D. Fischer, G. Irmer, *Appl. Spectrosc.* **2006**, *60*, 130.
[7] E. Garand, T. Wende, D. J. Goebbert, R. Bergmann, G. Meijer, D. M. Neumark, K. R. Asmis, *J. Am. Chem. Soc.* **2010**, *132*, 849.

[8] M. A. Moreno, B. Maté, Y. Rodríguez-Lazcano, O. Gálvez, P. C. Gómez, V. J. Herrero, R. Escribano, *J. Phys. Chem. A* **2013**, *117*, 9564.
[9] A. Stefánsson, K. H. Lemke, P. Bénézeth, J. Schott, *GCA* **2017**, *198*, 271.
[10] I. P. Gerothanassis, *Prog. Nucl. Magn. Reson. Spectrosc.* **2010**, *56*, 95.
[11] A. Wu, D. Cremer, J. Gauss, *J. Phys. Chem. A* **2003**, *107*, 8737.
[12] S. J. Clark, M. D. Segall, C. J. Pickard, P. J. Hasnip, M. I. J. Probert, K. Refson, M. C. Payne, *Z. Kristallogr. Cryst. Mater.* **2005**, *220*, 567.
[13] C. J. Pickard, F. Mauri, *Phys. Rev. B* **2001**, *63*, 245101.
[14] J. R. Yates, C. J. Pickard, F. Mauri, *Phys. Rev. B* **2007**, *76*, 024401.
[15] C. Bonhomme, C. Gervais, F. Babonneau, C. Coelho, F. Pourpoint, T. Azais, S. E. Ashbrook, J. M. Griffin, J. R. Yates, F. Mauri, et al., *Chem. Rev.* **2012**, *112*, 5733.
[16] D. Denysenko, M. Grzywa, M. Tonigold, B. Streppel, I. Krkljus, M. Hirscher, E. Mugnaioli, U. Kolb, J. Hanss, D. Volkmer, *Chem. Eur. J.l* **2011**, *17*, 1837.
[17] D. R. Allan, W. G. Marshall, C. R. Pulham, *Am. Mineral.* **2007**, *92*, 1018.
[18] Y. Duan, D. R. Luebke, H. W. Pennline, B. Li, M. J. Janik, J. W. Halley, *J. Phys. Chem. C* **2012**, *116*, 14461.
[19] K. J. Pike, V. Lemaitre, A. Kukol, T. Anupõld, A. Samoson, A. P. Howes, A. Watts, M. E. Smith, R. Dupree, *J. Phys. Chem. B* **2004**, *108*, 9256.
[20] I. Batatia, D. P. Kovacs, G. Simm, C. Ortner, G. Csanyi, *Adv. Neural Inf. Process Syst.* **2022**, *35*, 11423.
[21] D. P. Kovács, I. Batatia, E. S. Arany, G. Csányi, *J. Chem. Phys.* **2023**, *159*, 044118.
[22] L. L. Schaaf, E. Fako, S. De, A. Schäfer, G. Csányi, *npj Computational Materials* **2023**, *9*, 180.
[23] I. Batatia, P. Benner, Y. Chiang, A. M. Elena, D. P. Kovács, J. Riebesell, X. R. Advincula, M. Asta, M. Avaylon, W. J. Baldwin, F. Berger, N. Bernstein, A. Bhowmik, S. M. Blau, V. Cărare, J. P. Darby, S. De, F. D. Pia, V. L. Deringer, R. Elijošius, Z. El-Machachi, F. Falcioni, E. Fako, A. C. Ferrari, A. Genreith-Schriever, J. George, R. E. A. Goodall, C. P. Grey, P. Grigorev, S. Han, W. Handley, H. H. Heenen, K. Hermansson, C. Holm, J. Jaafar, S. Hofmann, K. S. Jakob, H. Jung, V. Kapil, A. D. Kaplan, N. Karimitari, J. R. Kermode, N. Kroupa, J. Kullgren, M. C. Kuner, D. Kuryla, G. Liepuoniute, J. T. Margraf, I.-B. Magdău, A. Michaelides, J. H. Moore, A. A. Naik, S. P. Niblett, S. W. Norwood, N. O'Neill, C. Ortner, K. A. Persson, K. Reuter, A. S. Rosen, L. L. Schaaf, C. Schran, B. X. Shi, E. Sivonxay, T. K. Stenczel, V. Svahn, C. Sutton, T. D. Swinburne, J. Tilly, C. van der Oord, E. Varga-Umbrich, T. Vegge, M. Vondrák, Y. Wang, W. C. Witt, F. Zills, G. Csányi, 'A foundation model for atomistic materials chemistry', arXiv - this paper is currently a pre-print, https://doi.org/10.48550/arXiv.2401.00096 (accessed 2024-07-31) **2024**.
[24] R. L. Vold, G. L. Hoatson, *J. Magn. Reson.* **2009**, *198*, 57.
[25] S. Sturniolo, T. F. G. Green, R. M. Hanson, M. Zilka, K. Refson, P. Hodgkinson, S. P. Brown, J. R. Yates, *Solid State Nucl. Magn. Reson.* **2016**, *78*, 64.
[26] A. P. M. Kentgens, *Geoderma* **1997**, *80*, 271.
[27] J. Struppe, S. Steuernagel, F. Aussenacc, F. Benevelli, P. Gierth, S. Wegner, *Bruker: Solid State NMR - AVANCE Solids User Manual. v3*, Bruker **2016**.
[28] D. Massiot, F. Fayon, M. Capron, L. C. King, I., B. S., Alonso, J.-O. Durand, B. Bujoli, Z. Gan, G. Hoatson, *Magn. Reson. Chem.* **2002**, *40*, 70.
[29] S. G. J. van Meerten, W. M. J. Franssen, A. P. M. Kentgens, *J. Magn. Reson.* **2019**, *301*, 56.
[30] M. Dračínský, P. Hodgkinson, *CrystEngComm* **2013**, *15*, 8705.
[31] M. Dračínský, P. Bouř, P. Hodgkinson, *J. Chem. Theory Comput.* **2016**, *12*, 968.
[32] O. Socha, P. Hodgkinson, C. M. Widdifield, J. R. Yates, M. Dračínský, *J. Phys. Chem. A* **2017**, *121*, 4103.
[33] R. Bjornsson, H. Früchtl, M. Bühl, *Phys. Chem. Chem. Phys.* **2011**, *13*, 619.
[34] V. Diez-Gómez, P. L. de Andres, J. Sanz, *ChemSusChem* **2020**, *13*, 1027.
[35] A. H. Berge, S. M. Pugh, M. I. M. Short, C. Kaur, Z. Lu, J.-H. Lee, C. J. Pickard, A. Sayari, A. C. Forse, *Nat. Commun.* **2022**, *13*, 7763.
[36] S. M. Pugh, A. C. Forse, *J. Magn. Reson.* **2023**, *346*, 107343.
[37] W. Makulski, K. Jackowski, *J. Magn. Reson.* **2020**, *313*, 106716.
[38] C. E. Bien, Q. Liu, C. R. Wade, *Chem. Mater.* **2020**, *32*, 489.
[39] R. A. Smaldone, R. S. Forgan, H. Furukawa, J. J. Gassensmith, A. M. Z. Slawin, O. M. Yaghi, J. F. Stoddart, *Angew. Chem. Int. Ed.* **2010**, *49*, 8630.
[40] D. C. Apperley, R. K. Harris, P. Hodgkinson, *Solid-State NMR : Basic Principles and Practice*, Momentum Press, New York, UNITED STATES **2012**.
[41] A. C. Forse, J. M. Griffin, C. P. Grey, *Solid State Nucl. Magn. Reson.* **2018**, *89*, 45.
[42] L. B. Alemany, D. M. Grant, R. J. Pugmire, T. D. Alger, K. W. Zilm, *J. Am. Chem. Soc.* **1983**, *105*, 2142.
[43] A. Peach, N. Fabregue, C. Erre, T.-X. Métro, D. Gajan, F. Mentink-Vigier, F. Scott, J. Trébosc, F. Voron, N. Patris, C. Gervais, D. Laurencin, *ChemRxiv, Preprint, doi:10.26434/chemrxiv-2024-04r36* **2024**.

[44] C. Odin, *Magn. Reson. Chem.* **2004**, *42*, 381.
[45] S. Takasaka, Y. Tsujimi, T. Yagi, *Phys. Rev. B* **1997**, *56*, 10715.
[46] S. Takasaka, Y. Tsujimi, T. Yagi, *Phys. Rev. B* **2002**, *65*, 174102.
[47] A. P. M. Kentgens, R. Verhagen, *Chem, Phys. Lett.* **1999**, *300*, 435.
[48] D. Iuga, H. Schäfer, R. Verhagen, A. P. M. Kentgens, *J. Magn. Reson.* **2000**, *147*, 192.
[49] M. E. Smith, S. Steuernagel, H. J. Whitfield, *Solid State Nucl. Magn. Reson.* **1995**, *4*, 313.
[50] M. Leskes, A. J. Moore, G. R. Goward, C. P. Grey, *J. Phys. Chem. C* **2013**, *117*, 26929.
[51] M. T. Dunstan, J. M. Griffin, F. Blanc, M. Leskes, C. P. Grey, *J. Phys. Chem. C* **2015**, *119*, 24255.
[52] M. Nava, N. Lopez, P. Müller, G. Wu, D. G. Nocera, C. C. Cummins, *J. Am. Chem. Soc.* **2015**, *137*, 14562, doi: 10.1021/jacs.5b08495.
[53] M. Bak, J. T. Rasmussen, N. C. Nielsen, *J. Magn. Reson.* **2000**, *147*, 296.
[54] K. Momma, F. Izumi, *J. Appl. Crystallog.* **2011**, *44*, 1272.
[55] B. M. Gatehouse, D. J. Lloyd, *J. Chem. Soc., Dalton Trans.* **1973**, pages 70–72.
[56] J. M. S. Skakle, M. Wilson, J. Feldmann, *Acta Crystallogr., Sect. E* **2001**, *57*, i94.
[57] A. M. Wright, Z. Wu, G. Zhang, J. L. Mancuso, R. J. Comito, R. W. Day, C. H. Hendon, J. T. Miller, M. Dincă, *Chem* **2018**, *4*, 2894.
[58] H. A. Patel, T. Islamoglu, Z. Liu, S. K. M. Nalluri, A. Samanta, O. Anamimoghadam, C. D. Malliakas, O. K. Farha, J. F. Stoddart, *J. Am. Chem. Soc.* **2017**, *139*, 11020.
